# Deciphering the ammonia transformation mechanism of a novel marine multi-stress-tolerant yeast, *Pichia kudriavzevii* HJ2, as revealed by integrated omics analysis

**DOI:** 10.1128/aem.02211-24

**Published:** 2025-05-08

**Authors:** Kunmei Huang, Huashan Bai, Can Meng, Muhammad Kashif, Zhiling Wei, Zaihang Tang, Shu He, Shanguang Wu, Sheng He, Chengjian Jiang

**Affiliations:** 1 Guangxi Key Laboratory for Green Processing of Sugar Resources, Liuzhou Key Laboratory of Guizhong Characteristic Medicinal Resources, College of Biological and Chemical Engineering, Guangxi University of Science and Technology 66514, Liuzhou, China; 2 State Key Laboratory for Conservation and Utilization of Subtropical Agro-bioresources, Guangxi Research Center for Microbial and Enzyme Engineering Technology, College of Life Science and Technology, Guangxi University 622308 , Nanning, China; 3 National Engineering Research Center for Non-Food Biorefinery, Guangxi Research Center for Biological Science and Technology, Guangxi Academy of Sciences 245477, Nanning, China; 4 Guangxi Birth Defects Prevention and Control Institute, Maternal and Child Health Hospital of Guangxi Zhuangzu Autonomous Region 597890 , Nanning, China; Chalmers tekniska hogskola AB, Gothenburg, Sweden

**Keywords:** *Pichia kudriavzevii *HJ2, ammonia nitrogen, biotransformation, omics analysis, amino acids

## Abstract

Ammonia nitrogen posed a significant threat to aquatic animals in aquaculture environments, and the substantial potential of microorganisms in removing ammonia nitrogen had garnered considerable attention. This study identified a marine yeast, *Pichia kudriavzevii* HJ2, which effectively removed ammonia nitrogen. By combining transcriptomics and metabolomics, the ammonia nitrogen transformation mechanism of HJ2 was elucidated. HJ2 achieved 100% ammonia nitrogen removal efficiency within 1 day of fermentation at 35°C with 300 mg/L ammonia nitrogen and 73.56% removal efficiency within 36 h with 600 mg/L ammonia nitrogen. Transcriptomics revealed that exposure to 600 mg/L ammonia nitrogen resulted in 541 up-regulated genes and 567 down-regulated genes in the HJ2 strain. Differentially expressed genes (DEGs) were primarily involved in the tricarboxylic acid (TCA) cycle and amino acid metabolism. Metabolomics revealed that HJ2 facilitated the production of 383 up-regulated metabolites and suppressed 137 down-regulated metabolites when exposed to 600 mg/L ammonia nitrogen. Integrating transcriptomics and metabolomics analyses showed that HJ2 removed ammonia nitrogen by sensing its presence in the extracellular environment, activating the TCA cycle, enhancing amino acid metabolism and nucleotide metabolism, and promoting its robust growth and reproduction. Amino acid metabolism played an important role in the ammonia transformation mechanism of HJ2. The result was confirmed by the increased activity of glutamate dehydrogenase (GDH) and aspartate aminotransferase (GOT). Up-regulated nitrogen metabolites such as L-glutamate, L-aspartic acid, spermidine, and trigonelline were produced. The results of enzyme activity tests, construction of overexpressing strains, and adding exogenous amino acid experiments demonstrated that HJ2 could utilize GDH and GOT ammonia assimilation pathways.

**IMPORTANCE:**

Ammonia nitrogen removal ability was a universal characteristic among the ammonia-oxidizing bacteria or archaea. Recently, yeast strains from the genus *Pichia* were found to have ammonia nitrogen removal ability. However, the mechanism of ammonia nitrogen removal in *Pichia* had not been reported. In the study, the ammonia nitrogen removal efficiency of *Pichia kudriavzevii* HJ2 was identified, and the mechanisms by which HJ2 transformed ammonia nitrogen into non-toxic organic nitrogen were elucidated, offering potential solutions to pollution challenges in aquaculture and helping minimize resource waste. The study offered new insights into the transformation mechanism of microbial ammonia nitrogen removal and its environmentally friendly application.

## INTRODUCTION

The removal of inorganic nitrogenous compounds (e.g., NH_4_^+^ and NH_3_) in aquatic ecology had been a research hotspot (1, 2). Unionized ammonia (NH_3_), which was highly toxic due to its fat solubility, could penetrate cell membranes and cause detrimental effects on the economic value of aquatic animals (3). The diffusion of ammonia nitrogen into cells could disrupt amino acid (AA) metabolism in aquatic animals, result in oxidative stress, immunosuppression, and potentially death (4). Additionally, excessive ammonia nitrogen levels could cause explosive plankton growth, such as algae, and disrupt aquatic ecological structures (5). Thus, the removal of ammonium nitrogen (NH_3_/NH_4_^+^) had garnered increased interest across various aquacultural ecosystems, including domestic sewage treatment, agricultural runoff management, and industrial effluent control (6).

Recently, physical, chemical, and biological methods had been employed to remove ammonia nitrogen (7). However, physical and chemical methods incurred significantly higher costs and posed potential risks in terms of ecological toxicity. Microbial technology had gained considerable interest as a favorable strategy due to its environmentally friendly and sustainable nature (8). Various bacteria, including *Methanothermobacter* sp. DTU77*9* (9), *Bacillus velezensis* LG37 (10), and *Bacillus subtilis* NX-2 (11), possessed functional capabilities to remove ammonia nitrogen. Fungi such as *Saccharomyces cerevisiae* (12), *Sporidiobolus pararoseus* Y1 (13), and *P. kudriavzevii* GW1 (14) were also identified.

Microorganisms, particularly ammonia-oxidizing archaea, had developed various strategies to remove ammonia nitrogen. These strategies involved ammonium transporters, ammonia oxidation, and assimilation pathways that utilized glutamate dehydrogenase (GDH) or the glutamine synthetase–glutamate synthase (GS/GOGAT) system (15).

Mep1 and Mep2 were high-affinity transporters for NH_4_^+^ uptake in bacteria and fungi (16, 17). In *Candida albicans*, CaMEP1 was associated with NH_4_^+^ transport, whereas CaMEP2 was involved in electroneutral NH_3_ transport (18). The transmembrane transport of electroneutral NH_3_ in *S. cerevisiae* was facilitated by ScMep2, which was regulated by cytoplasmic pH (19). Additionally, some researches had revealed that Mep2 was regulated by Npr1 kinase and functions as a crucial receptor in signal transduction processes (20, 21). However, the role of ammonia transport in *P. kudriavzevii* remained poorly reported.

Intracellular NH_4_^+^ could undergo denitrification through aerobic/heterotrophic nitrification and denitrification for ammonia nitrogen removal. Ammonia was initially oxidized to nitrite by ammonia-oxidizing bacteria or archaea and subsequently converted to nitrate by nitrite-oxidizing bacteria (22). Alternatively, ammonia oxidation could be accomplished in a single step through comammox (23). Through chemical and omics analyses, an alternative pathway for ammonia nitrogen removal was discovered in the oligotrophic HNAD strain of *Pseudomonas* sp. N31942. In the pathway, NH_4_^+^-N could be directly eliminated in GDH/GS-GOGAT through a chemical process (24). In yeast strains lacking GDH, proline utilization (PUT) or the GS/GOGAT pathway could be an alternative pathway for glutamate production and nitrogen assimilation (25). Thus, GDH also played a key role in glutamate biosynthesis and nitrogen assimilation. Furthermore, ammonia nitrogen facilitated the synthesis of amino acids in ruminal microbiota through ammonia assimilation. Supplementation of amino acids at various concentrations resulted in an augmented production of branched-chain volatile fatty acids, which were subsequently incorporated into microbial proteins and other intracellular macromolecules (26). Converting ammonia nitrogen into non-toxic organic nitrogen also contributed to reducing aquaculture pollution and resource waste (27).

*P. kudriavzevii* had been demonstrated to possess a remarkable capacity for removing ammonia nitrogen and producing acid (14, 28). However, few reports had specifically addressed the ammonia nitrogen removal efficiency of *P. kudriavzevii*. In a previous study, *P. kudriavzevii* HJ2, isolated from a subtropical marine mangrove environment, exhibited potential probiotic characteristics and was proved safe in specific-pathogen-free (SPF) healthy adult mice (29). The present study focused on investigating the efficiency of ammonia nitrogen removal by HJ2 through single-factor and orthogonal optimization strategies. The growth curve and cell morphology of HJ2 were investigated during ammonia nitrogen removal. Furthermore, transcriptome and metabolome analyses were employed to elucidate the underlying mechanism of ammonia nitrogen transformation by HJ2. The ammonia assimilation pathways in HJ2 were confirmed through enzyme activity tests and the construction of overexpressed strains. The study provided a scientific foundation for the further environmentally friendly application of ammonia nitrogen wastewater treatment in aquaculture.

## RESULTS

### Physicochemical and morphological characteristics of ammonia nitrogen removal by HJ2

#### Single-factor experiments and orthogonal test

**Fig 1:**
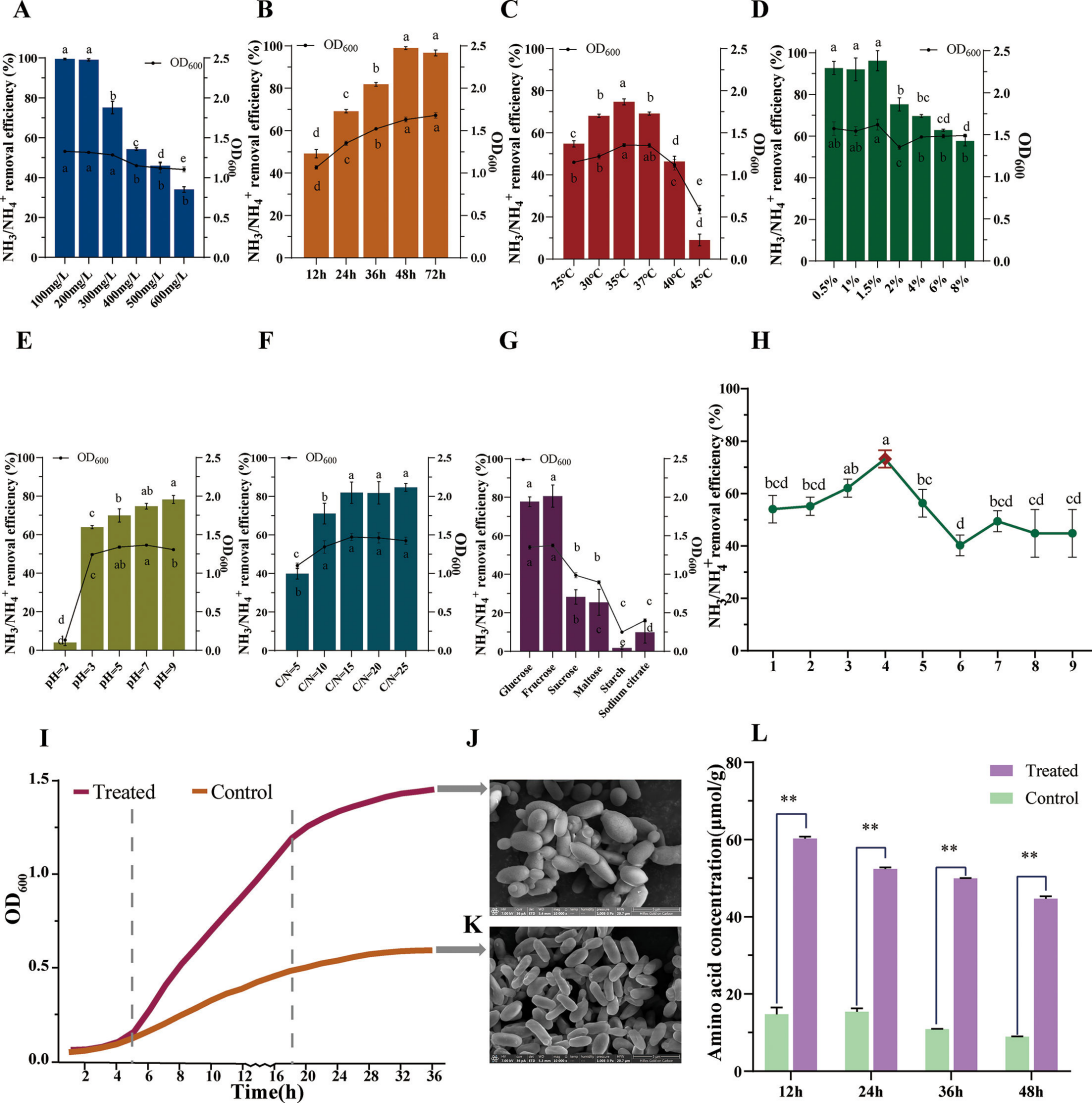
Physicochemical characteristics and morphology of ammonia nitrogen removal efficiency in *P. kudriavzevii* HJ2. Ammonia nitrogen removal efficiency of HJ2 at different (A) ammonia nitrogen concentrations, (B) times, (C) temperatures, (D) inoculation concentrations, (E) pH values, (F) C/N ratios, and (G) carbon sources. The left *y*-axis represented the ammonia nitrogen removal efficiency, and the right *y*-axis represented the OD_600_ of HJ2 in the single-factor experiments. (**H**) Dot diagram of orthogonal experimental results. Above data were expressed as mean ± SD. All error bars indicated the standard error of the means for biological triplicates. *P* values were calculated by one-way ANOVA. Significance was represented by lowercase letters. (**I**) HJ2 growth curves of the control group and treated group based on orthogonal optimized culture conditions. Imaginary lines indicated different growth phases in *P. kudriavzevii* HJ2 under 0 and 600 mg/L ammonia nitrogen. Lag phase, 0–5 h; exponential growth phase, 5–18 h; stationary phase: after 18 h. (**J**) Scanning electron microscopy observation of the treated group. Bar = 5 µm. (**K**) Scanning electron microscopy observation of the control group. Bar = 5 µm. (**L**) Total amino acid content of different time in the control group and treated group of HJ2. The data were expressed as mean ± SD. All error bars indicated the standard error of the means for biological triplicates. *P* values were calculated by *t*-test. *P values *<* 0.05. **P values *<* 0.01.

HJ2 was cultured for 24 h at various concentrations of ammonia nitrogen ranging from 100 mg/L to 600 mg/L and yielded removal efficiencies of 100%, 100%, 75.16%, 54.31%, 45.98%, and 34.06%, respectively, as shown in Fig. 1A. Extending the fermentation time to 48 h and 72h increased the ammonia nitrogen removal efficiency to 100% under 300 mg/L ammonia nitrogen (Fig. 1B). Microorganisms inhabited environments where water temperature fluctuated seasonally. Water temperature variations significantly influenced the metabolic activity of microbial enzymes. Therefore, investigating the impact of temperature on the characteristics of nitrogen-removing microorganisms served as a crucial guide for managing water quality (30). The conversion of ammonia nitrogen was observed to be less efficient at 25°C. However, between 30°C and 37°C, HJ2 exhibited a gradual increase in its capacity to transform ammonia nitrogen, reaching its peak level of 74.78% at 35°C. Subsequently, as the temperature reached 40°C, the removal capacity for ammonia nitrogen gradually decreased. The optimization of water temperature regulation appropriately improved the performance of HJ2 in removing ammonia nitrogen, as shown in Fig. 1C. Low inoculation concentration prolonged the culture time, negatively impacting the removal efficiency of ammonia nitrogen. Conversely, an excessively high inoculation concentration could result in insufficient dissolved oxygen, adversely affecting product synthesis and increasing metabolic intermediate accumulation. Therefore, the inoculation concentration was also an important factor in water quality treatment (11). At an inoculation concentration ranging from 0.5% to 1.5%, the ammonia nitrogen removal efficiency reached 100%. However, the ability exhibited a significant decline beyond an inoculation concentration of 1.5% (Fig. 1D). The result showed that HJ2 demonstrated superior ammonia nitrogen removal efficiency at low inoculation concentration levels and high survival efficiency compared with strains with high inoculation concentration levels. Improper pH value would damage microbial activity. Different strains had different ammonia nitrogen removal abilities at different pH values (31). The ammonia nitrogen removal capacity of HJ2 was found to be the lowest at only 3.95% at pH 2.0. However, the strain demonstrated high proficiency in transforming ammonia nitrogen across a pH range of 3.0–9.0, indicating a broad pH range of applicability (Fig. 1E). The carbon-to-nitrogen ratio (C/N) affected microbial growth and energy conversion, serving as an essential parameter for assessing electron donor and receptor requirements (32). When cultured at a C/N of 5 for 24 h, HJ2 also exhibited an ammonia nitrogen removal efficiency of 39.84% (Fig. 1F). When the C/N was increased to 10, the ammonia nitrogen removal efficiency of HJ2 further increased. The ammonia nitrogen removal efficiency did not differ significantly among C/N ≥ 15. However, C/N of 15 was found to be particularly optimal for ammonia nitrogen removal in the HJ2 strain. Carbon sources were important electron donors for strain growth and nitrogen metabolism, and the carbon metabolism and nitrogen metabolism were fundamental cellular metabolic pathway in biological water treatment technologies (33). Adding the suitable carbon source could increase the removal efficiency of ammonia nitrogen. The strain utilized monosaccharides (glucose and fructose) as its sole carbon source, resulting in the highest efficiency of ammonia nitrogen removal. It was followed by disaccharides (sucrose and maltose), polysaccharides (starch), and organic acid (sodium citrate), as shown in Fig. 1G.

As shown in Fig. 1B, the growth index (OD_600_) of HJ2 cells exhibited a significant increase at the same ammonia nitrogen concentration, and the removal efficiency of ammonia nitrogen was significantly improved. This finding was consistent with the results of Xie et al., who reported that the effective removal of ammonia nitrogen by strain H1 was accompanied by an increase of cell growth index (34). The trends observed under varying temperatures (Fig. 1C), pH 2–7 (Fig. 1E), C/N 5–15 (Fig. 1F), and different carbon sources (Fig. 1G) exhibited a similar pattern. Specifically, the ammonia nitrogen removal efficiency increased with the growth index increasing in the HJ2 strain. However, HJ2 did not show an obvious similar trend under varying ammonia nitrogen concentrations (Fig. 1A), different inoculation amounts (Fig. 1D), pH 9.0 (Fig. 1E), and C/N 20–25 (Fig. 1F). These findings suggested that specific environmental factors could influence both cell growth and removal efficiency of ammonia nitrogen. At the same ammonia nitrogen concentrations, changes in pH, temperature, C/N, and carbon sources could significantly impact cell growth, which in turn affected the efficiency of ammonia nitrogen removal under various conditions. An unsuitable environment could impair cell growth, ultimately leading to a decrease in ammonia nitrogen removal efficiency. Therefore, environmental factors were correlated with both cell growth and ammonia nitrogen removal efficiency.

The orthogonal optimization analysis of ammonia nitrogen removal efficiency indicated that culture time exerted the most significant influence, followed by culture temperature, whereas inoculation concentration exhibited a relatively minor impact. HJ2 exhibited the highest removal efficiency for high ammonia nitrogen (600 mg/L) under the following conditions: a culture temperature of 35°C, an inoculation concentration of 0.5%, and a culture time of 36 h, with a recorded value of 73.56% (Fig. 1H).

#### Growth morphological analysis of ammonia nitrogen removal of HJ2

The growth curves of HJ2 cultured in the control group and treated group were shown in Fig. 1I. The lag phase, logarithmic growth phase, and stationary phase of HJ2 were 0-5 h, 5-18 h, and 18-36 h, respectively. Notably, the maximum OD_600_ value of the control group was 0.62, whereas that of the treated group reached 1.45, which was 2.34 times higher than that of the control group. Previous studies speculated that ammonia nitrogen was converted into protein and other nutrients, supporting cell growth and proliferation in HJ2 (24, 35). Cell morphology was observed by scanning electron microscopy (Fig. 1J). In the control group, HJ2 cells appeared short and slightly shriveled, with lengths ranging from 2.57 to 3.00 µm, and diameters from 1.14 to 1.43 µm (Fig. 1K). Conversely, cells in the treated group were notably larger, fuller, and ellipsoidal, measuring from 3.14 to 4.29 µm in length and 1.86 to 2.86 µm in diameter-approximately twice the size of those in the control group (Fig. 1J). Additionally, the treated group exhibited a higher germination rate, with cells showing increased germination and vigorous growth compared with the control group. The growth curve as shown in Fig. 1I further indicated that HJ2 cells exhibited rapid growth, enhanced reproduction, increased cell size, and elevated metabolic activity under high ammonia nitrogen conditions. These findings suggested that HJ2 cells effectively absorbed and utilized ammonia nitrogen under high ammonia nitrogen treatment, thereby promoting robust cell growth.

#### Intracellular total AA content of HJ2 under ammonia nitrogen treatment

Amino acids, fundamental units for protein synthesis, could be utilized for synthesizing various substances such as nucleic acids (36). The total AA content of the HJ2 wet cell in the treated group decreased over time, with values of 60.25 µmol/g at 12 h and 44.73 µmol/g at 48 h. These values were significantly higher than those in the control group at the same time points (*P* < 0.01), with increases of approximately 4.09-fold and 5.00-fold, respectively (Fig. 1L). Additionally, the AA content in the treated group was significantly higher than in the control group at all time points (*P* < 0.01). The concentration of intracellular amino acids gradually decreased over time (Fig. 1L). As shown in Fig. 1B, the efficiency of ammonia nitrogen removal increased with time, suggesting that during ammonia nitrogen removal, HJ2 assimilated extracellular ammonia nitrogen and subsequently converted it into intracellular amino acids. These amino acids served as crucial precursors for protein synthesis and the biosynthesis of proteins, nucleotides, and other macromolecules (37). These findings speculated that HJ2 effectively transformed ammonia nitrogen into intracellular products during ammonia nitrogen removal.

### Transcriptome analysis of ammonia nitrogen transformation mechanism of *P. kudriavzevii* HJ2

#### Differential expression genes, functional annotation analysis, and RT-qPCR

**Fig 2:**
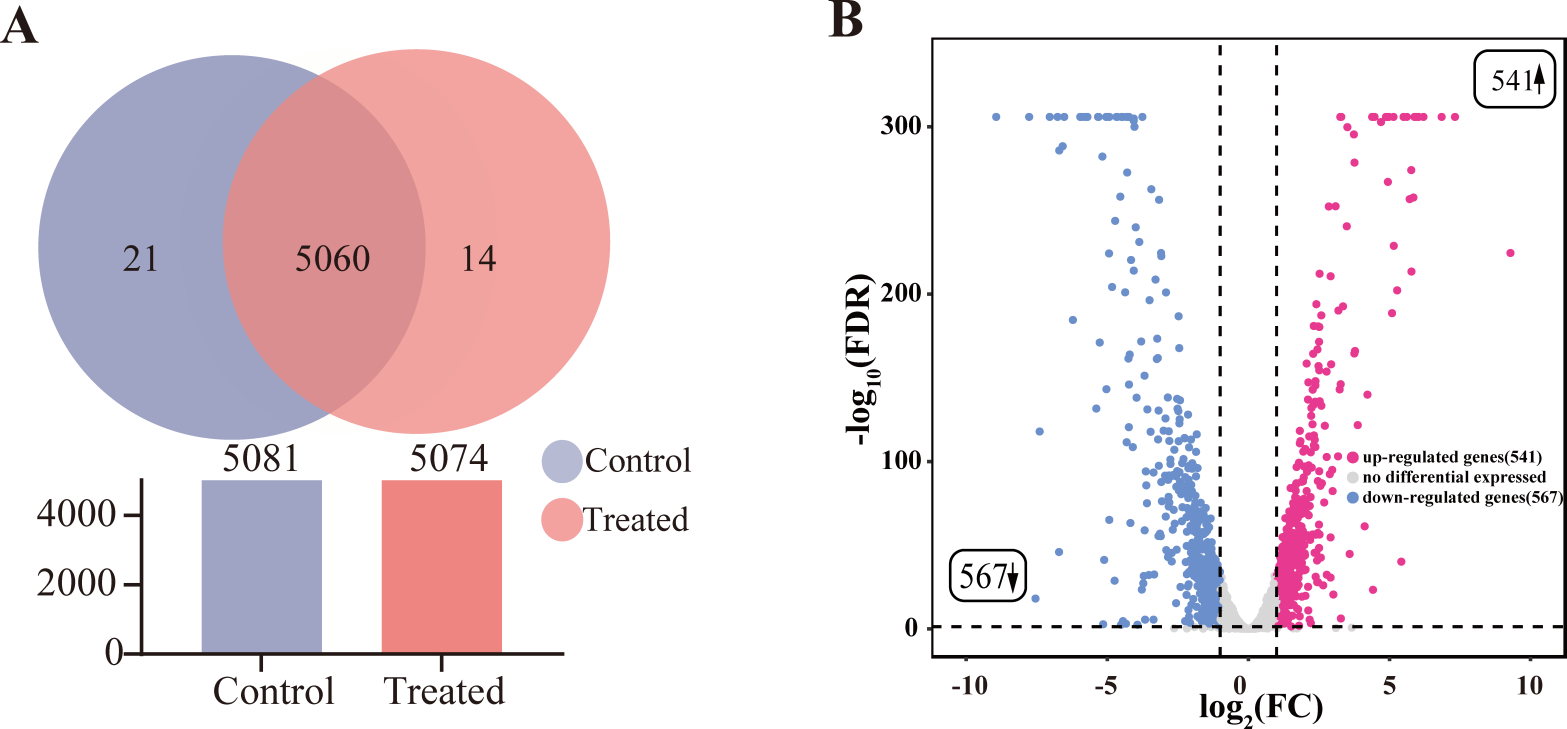
Expression proﬁles of DEGs in *P. kudriavzevii* HJ2 were incubated at different ammonia nitrogen conditions. (**A**) The Venn diagrams of the genes in HJ2 on the left side show that the total number of co-expressed genes in the control group and the treated group was 5,060. The number of genes expressed only in the control group was 21. The number of genes expressed only in the treated group was 14. (**B**) Volcano plot showing DEGs on the right side with 541 up-regulated genes (*P*_adj_ < 0.05, log_2_ fold change ≥ 1) in red and on the left side with 567 down-regulated genes (*P*_adj_ < 0.05, log_2_ fold change ≤ –1) in blue in *P. kudriavzevii* HJ2 incubated at 600 mg/L ammonia nitrogen. Genes without significant differential expression between the control group and treated group were in gray.

The transformation mechanism of ammonia nitrogen removal in HJ2 was investigated by extracting total RNA and sequencing mRNA. The Q20, Q30, raw reads, clean reads, and GC content of each sample in the control groups and treated groups were presented in Table S1. Transcriptome analysis revealed 1,108 DEGs (*P*_adj_ < 0.05, |log_2_ fold change| ≥1) between the control group and the treated group, including 541 up-regulated genes and 567 down-regulated genes, as shown in Fig. 2A and B. Seven DEGs were selected for RT-qPCR. The results showed a slight change in the expression of these DEGs but similar expression trends, confirming the reliability of transcriptome availability. The primers used in RT-qPCR and the results of RT-qPCR were depicted in Table S2 and Fig. S1, respectively.

In terms of the Kyoto Encyclopedia of Genes and Genomes (KEGG) pathway enrichment analysis, the up-regulated genes were predominantly significantly enriched in 16 significant pathways (Fig. S2A). These pathways included carbon metabolism (involving the glycolysis metabolism and tricarboxylic acid [TCA] cycle), biosynthesis of amino acids, and biosynthesis of secondary metabolites. Furthermore, the oxidative phosphorylation pathway played a crucial role in energy metabolism. Conversely, the down-regulated genes, as depicted in Fig. S2B, exhibited significant enrichment in 11 pathways, which included the ABC transporter pathways, fatty acid degradation, and autophagy. DEGs significantly enriched multiple KEGG pathways. Notably, we observed that the up-regulated genes were more abundant in the KEGG enrichment pathway compared with the down-regulated genes.

#### Transformation mechanism of HJ2 ammonia nitrogen removal by transcriptome analysis

**Fig 3:**
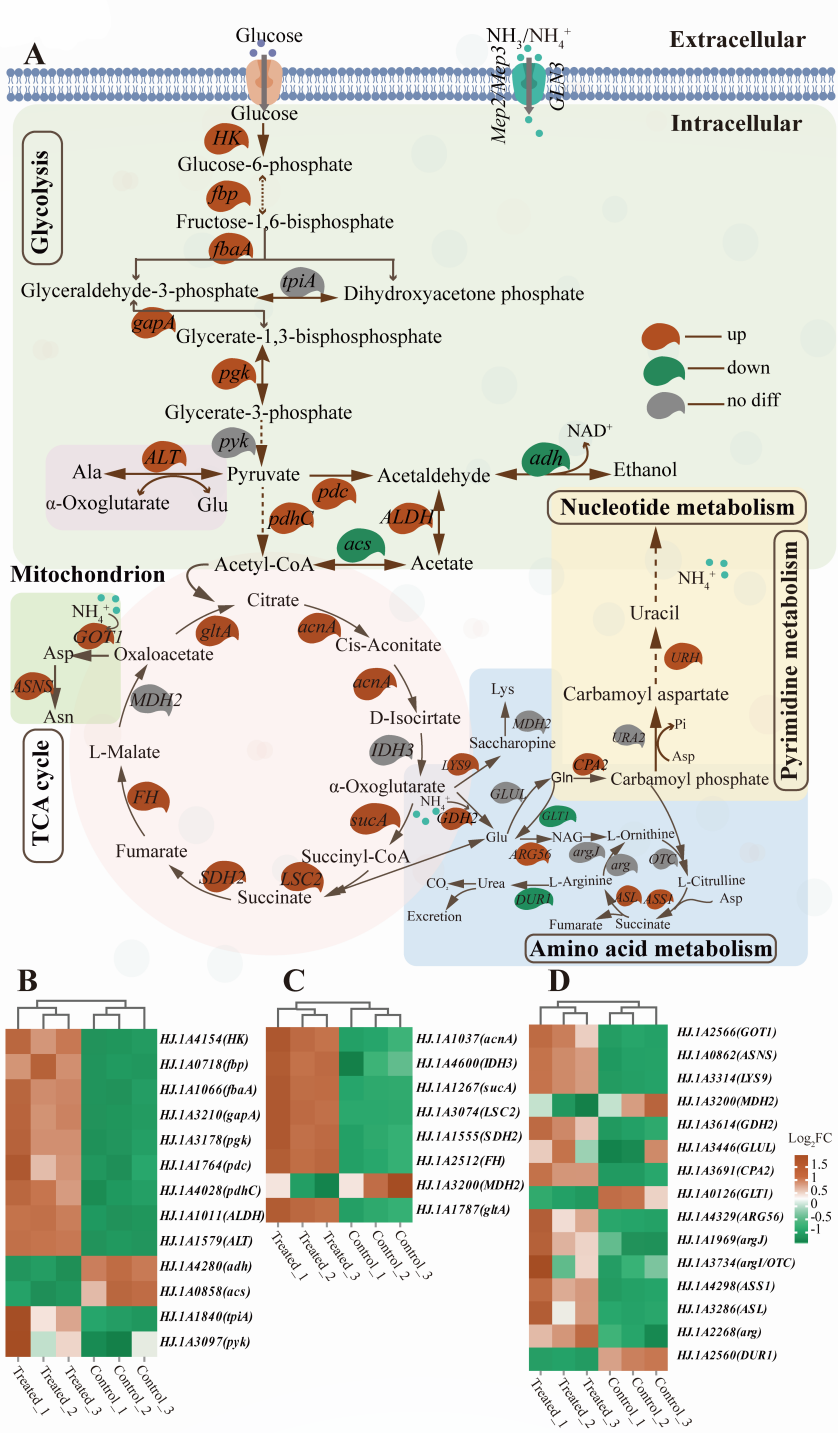
Transcriptomics revealed the differential genes expression level in *P. kudriavzevii* HJ2 incubated at 600 mg/L ammonia nitrogen. (**A**) Prediction of the ammonia nitrogen removal transformation mechanism in HJ2 by using transcriptomics. Up-regulated genes were shown in red background, down-regulated genes were in green background, and genes with no significant difference were in gray background. Genes were indicated in italics. For enzyme reactions, the arrows between two metabolites represented the directions of catalytic reactions. Solid arrows represented direct reactions, and dashed arrows indicated indirect reactions. Different regions of the color-coded plates represented distinct metabolic pathways. The green plate indicated the glycolysis pathway, the pink plate corresponded to the TCA cycle, the yellow plate denoted nucleotide metabolism, and the blue plate signified amino acid metabolism. (**B**) Heatmap displaying the expression levels of DEGs involved in the glycolysis and pyruvate metabolism pathways of the control group and treated group. (**C**) Heatmap depicting the expression level of DEGs involved in the TCA cycle of the control group and treated group. (**D**) Heatmap illustrating the expression level of DEGs involved in the amino acid metabolism in the control group and treated group. In panels B to D, the color blocks represented the normalized value of gene expression. Lines at the top of the color blocks indicated that the cluster analysis had been performed. The shorter the clustering branches, the higher the similarity. *P*_adj_ < 0.05 and log_2_ FC of ≥1 within the red block denoted an upregulated gene. *P*_adj_ < 0.05 and log_2_ FC of ≤–1 within the green block indicated a down-regulated gene. HK, hexokinase; fbp, fructose-1,6-bisphosphatase; fbaA, fructose-bisphosphate aldolase; gapA, glyceraldehyde-3-phosphate dehydrogenase; pgk, phosphoglycerate kinase; ALT, alanine aminotransferase; pdc, pyruvate decarboxylase isozyme; pdhC, pyruvate dehydrogenase; ALDH, aldehyde dehydrogenase; gltA, citrate synthase; acnA, aconitate hydratase; sucA, 2-oxoglutarate dehydrogenase; LSC2, succinyl-CoA synthetase; SDH2, succinate dehydrogenase; FH, fumarate hydratase; GOT1, aspartate transaminase; ASNS, asparagine synthase; LYS9, fumarase; GDH2, glutamate dehydrogenase; ARG56, N-acetyl-glutamate semialdehyde dehydrogenase; ASL, arginine succinate lyase; ASS1, argininosuccinate synthase; CPA2, carbamoyl phosphate synthase; URH, inosine-uridine preferring nucleoside hydrolase.

The transformation mechanism of ammonia nitrogen removal in HJ2 was investigated through transcriptome analysis (Fig. 3). Firstly, the glycolysis pathway and TCA cycle were essential metabolic pathways involved in glucose metabolism, as well as the biosynthesis of lipids, nucleotides, and amino acids. They played a significant role in cell growth and proliferation (38, 39). As shown in Fig. 3A and B, transcriptome analysis revealed that exposure of the HJ2 strain to high ammonia nitrogen significantly up-regulated genes associated with the glycolysis pathway. Specifically, ammonia nitrogen increased the up-regulated expression of glycolysis-related genes, including *HK, fbp, fbaA, gapA, pgk, pdhC, pdc, ALDH,* and *ALT*. These genes were involved in the production of precursors for the TCA cycle, such as acetyl-CoA. The results suggested that high ammonia nitrogen not only affected nitrogen metabolism but also redirected metabolic flux toward glycolysis, potentially enhancing the production of key intermediates required for cellular energy and biosynthetic processes. At the same time, the TCA cycle was significantly enriched. The up-regulated expression of the TCA cycle-related genes (such as *acnA, sucA, LSC2, SDH2, FH,* and *gltA*) in the HJ2 strain under high ammonia nitrogen conditions could lead to the biosynthesis of organic acids (citrate and L-malate) and precursors for amino acid metabolism (oxaloacetate and α-oxoglutarate). Organic acids could modulate the environmental pH toward acidity. Acidic environment could inhibit the deamination of organic nitrogen compounds, thereby facilitating the conversion of ammonia nitrogen into organic nitrogen compounds in the HJ2 strain (28, 40). Amino acids served as components of protein translation, substrates for nucleotide synthesis, and contributors to maintaining cellular redox balance (41). The synthesis of amino acids could be achieved by amino acid metabolism. The up-regulated genes related to amino acid metabolism (*GDH2, GOT1, ASNS, ALT, ASS1, ASL, LYS9,* and *ARG56*) enhanced the production of amino acids including L-glutamate, glutamine, and L-aspartic acid. The glutamine synthesis was an effective strategy to deal with ammonia toxicity in cells (3). The ammonium transporter gene was most down-regulated at high concentrations of ammonium (42). A previous study had suggested that high intracellular glutamine levels could inhibit the ammonium transporter, likely through a feedback mechanism regulating nitrogen uptake and metabolism (43). The down-regulation of ammonium transporter genes and nitrogen regulator-related genes (*Mep2*, *Mep3*, and *GAT1*) supported the conclusion that the level of intracellular glutamine was high in the HJ2 strain. Furthermore, nucleotide metabolism played a critical role in transmitting genetic information during cell growth. Aspartic acid and glutamine were key intermediates in nucleotide metabolism (44). Under ammonia nitrogen treatment, the up-regulated genes related to nucleotide metabolism (CPA2 and URH) in the HJ2 strain supported its growth and reproduction. These genes likely enhance nucleotide synthesis and recycling, which were essential for DNA and RNA production required during cell division and growth. Moreover, the up-regulated genes involved in oxidative phosphorylation (*ATP1, COX6, CYT1, SDH1,* and *QCR2*) ensured rapid oxidative phosphorylation metabolism and ATP production. This increased energy generation provides the necessary energy for cellular processes, including growth and maintenance, thereby facilitating the strain’s ability to thrive under ammonia nitrogen conditions (45) (Table S3). The significant enrichment of the cellular metabolic pathways in the HJ2 strain under ammonia nitrogen treatment demonstrated that it synthesizes intracellular macromolecules through various pathways, including carbon metabolism (glycolysis and the TCA cycle), amino acid metabolism, and oxidative phosphorylation. This metabolic flexibility promoted cell growth and reproduction while facilitating ammonia nitrogen transformation and removal.

### Metabolome analysis of HJ2

#### Analysis of metabolites in ammonia nitrogen treatment

**Fig 4:**
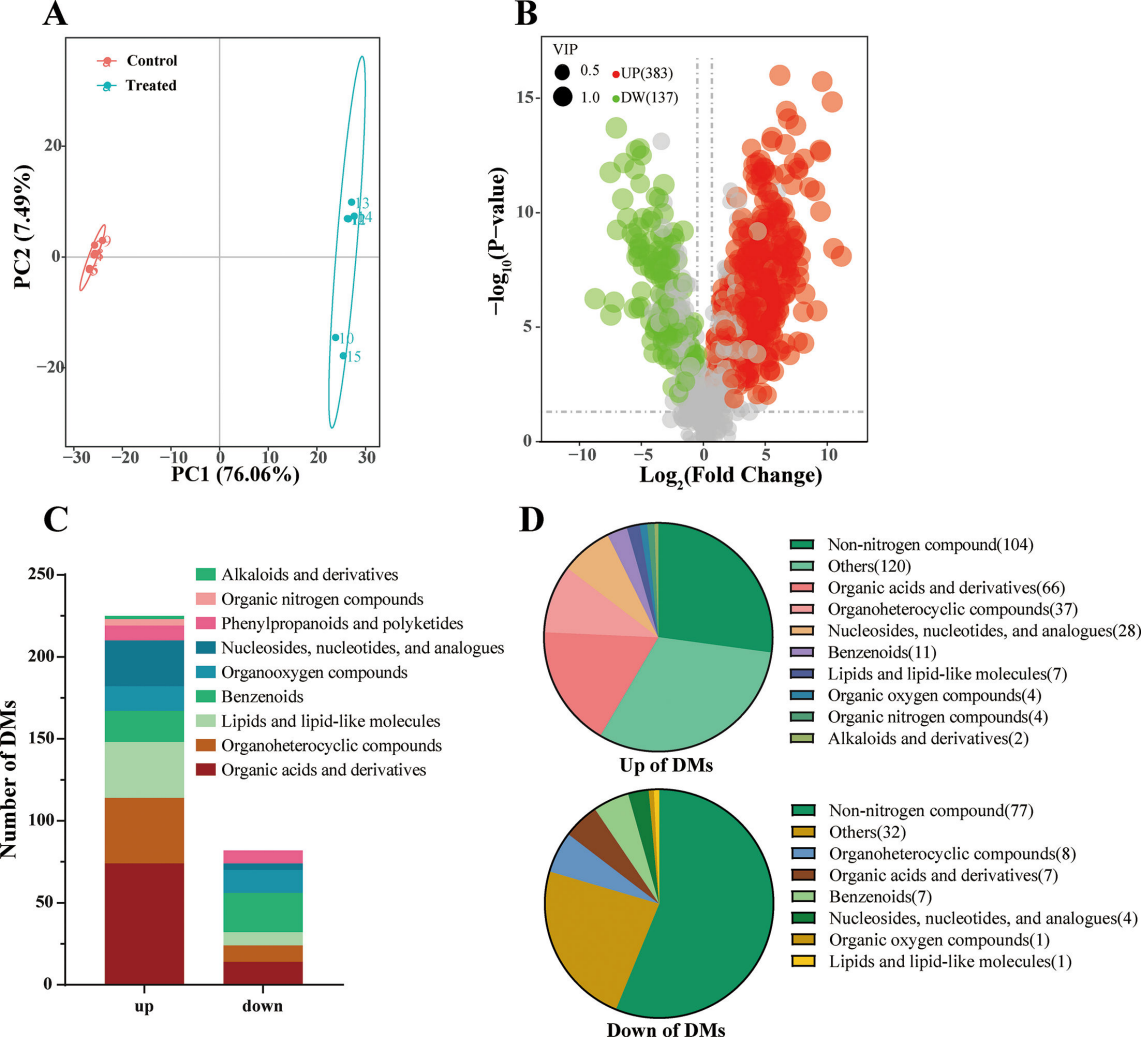
Metabolite analysis of the metabolome in *P. kudriavzevii* HJ2 incubated at 600 mg/L ammonia nitrogen. (**A**) PCA diagram. The red represented the control group. The blue represented the treated group. In this figure, abscissa PC1 and ordinate PC2 represented the scores of the first and second principal components, respectively. (**B**) Volcano plot showing the DMs. There were 383 up-regulated metabolites (*P*_adj_ < 0.05, log_2_ fold change ≥ 1) in red and 137 down-regulated metabolites (*P*_adj_ < 0.05, log_2_ fold change ≤ –1) in green in *P. kudriavzevii* HJ2 incubated at 600 mg/L ammonia nitrogen. Metabolites without significant difference between the control group and treated group were in gray. (**C**) HMDB classiﬁcation of DMs in nine categories: alkaloids and derivatives; organic nitrogen compounds; phenylpropanoids and polyketides; nucleosides, nucleotides, and analogs; organooxygen compounds; benzenoids; lipids and lipid-like molecules; organoheterocyclic compounds; and organic acids and derivatives. The *y*-axis indicated the number of DMs in a category. The *x*-axis indicated the categories of the up-regulated and down-regulated metabolites. (**D**) Pie chart of the proportion of non-nitrogen compounds and specific nitrogen compounds in DMs. The pie chart above depicted the distribution of non-nitrogen and specific nitrogen compounds in the up-regulated metabolites. The pie chart below illustrated the distribution of non-nitrogen and specific nitrogen compounds in the down-regulated metabolites.

To further elucidate the transformation mechanism of ammonia nitrogen removal in HJ2, we analyzed the metabolites of HJ2. Principal component analysis (PCA) was employed to identify differences in metabolites between the control group and treated group (Fig. 4A). Compared with the control group, the treated group exhibited a notable biological response, accompanied by significant changes in metabolites. A total of 520 differential metabolites (DMs) (VIP > 1, *P* < 0.05, |log_2_ FC| ≥1) were identified, including 383 up-regulated metabolites and 137 down-regulated metabolites, as shown in the volcano plot (Fig. 4B). Detailed information on the DMs detected by metabolomics is listed in Table S4. The number of up-regulated metabolites exceeded that of down-regulated metabolites. Nine categories were marked in the Human Metabolome Database (HMDB), including nucleosides, nucleotides, and analogs, organoheterocyclic compounds, and organic acids and derivatives (Fig. 4C). Up-regulated metabolites were mainly enriched in organic acids and their derivatives, peptides, and amino acids. Moreover, we observed an increase in organic heterocyclic compounds such as pyrrole carboxylic acid and its derivatives, as well as nucleotides and their analogs. The KEGG enrichment analysis of DMs s demonstrated in Fig. S3. The pathways related to amino acid metabolism and nucleotide metabolism were predominantly enriched among the upregulated DMs. These findings were consistent with the annotation provided by HMDB. The KEGG enrichment trend of metabolomics was similar to that of transcriptomics. Given the significant up-regulated metabolites in amino acid metabolism, we performed the KEGG enrichment analysis to investigate the enrichment of the up-regulated and down-regulated metabolites in amino acid metabolism. These results were presented in Fig. S4. The up-regulated metabolites were enriched in the pathways of arginine and proline metabolism, alanine metabolism, and glutathione metabolism. These results indicated that amino acid metabolism played an important role in ammonia nitrogen transformation in the HJ2 strain.

Furthermore, the DMs were categorized into non-nitrogen-containing DMs and nitrogen-containing DMs (N-DMs) in the study. The proportion of the up-regulated N-DMs accounted for 72.84% of the total up-regulated metabolites (279 out of 383). Conversely, the proportion of the down-regulated N-DMs accounted for 43.80% of the total down-regulated metabolites (60 out of 137), as shown in Fig. 4D. Notably, the up-regulated N-DMs was 4.65 times higher than the down-regulated N-DMs. From a metabolomic analysis perspective, the results suggested that ammonia nitrogen was transformed into N-DMs.

#### Analysis of metabolites by HPLC

**Fig 5:**
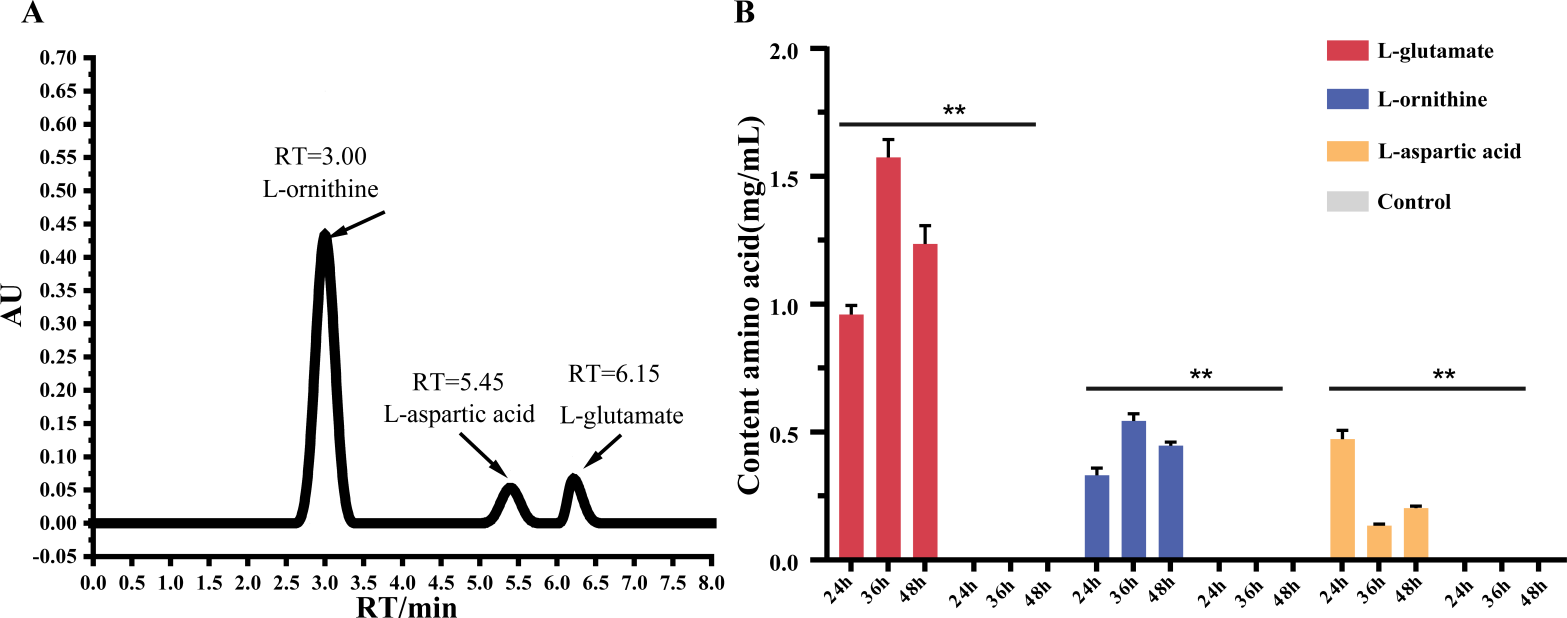
Content of L-glutamate, L-aspartic acid, and L-ornithine detected by HPLC. (**A**) Peak of L-ornithine, L-glutamate, and L-aspartic acid in HJ2 incubated at 600 mg/L ammonia nitrogen. The *y*-axis indicated the absorbance unit (AU) of L-ornithine, L-glutamate, and L-aspartic acid. The *x*-axis indicated the retention time (RT) of the peak of L-ornithine, L-glutamate, and L-aspartic acid. (**B**) Histogram of the contents of L-ornithine, L-glutamate, and L-aspartic acid in HJ2. The *y*-axis indicated the concentration of L-ornithine, L-glutamate, and L-aspartic acid. The *x*-axis represented the categories of L-ornithine, L-glutamate, and L-aspartic acid. The data were expressed as mean ± SD. The error bars indicated the standard error of the means for biological triplicates. *P* values were calculated by *t*-test. *P values *<* 0.05, **P values *<* 0.01.

Under ammonia nitrogen treatment, HJ2 produced a high concentration of free amino acids. Twelve kinds of amino acids were detected by metabolomic analysis:, D-glutamine, L-glutamate, L-aspartic acid, L-asparagine, L-histidine, L-lysine, L-methionine, L-tyrosine, L-valine, L-phenylalanine, D-phenylalanine, and L-ornithine (Table 1). L-glutamate had the highest concentration (log_2_ FC = 10.42). Additionally, we observed an increase in glutathione levels. Out of the nine types of common AAs identified, six AAs were determined to be essential, including L-histidine and L-tyrosine, collectively constituting 66.67% of the conventional AAs detected (37). These findings of metabolomics analysis were aligned with the anticipated up-regulated amino acid products, as indicated by transcriptomics analysis. The concentrations of L-glutamate, L-aspartic acid, and L-ornithine were determined by high-performance liquid chromatography (HPLC) (Fig. 5). When HJ2 was exposed to ammonia nitrogen, the content of L-glutamate (Glu) significantly surpassed that of L-aspartic acid (Asp) and L-ornithine (L-Orn) over time. After 36 h of ammonia nitrogen treatment, the content of L-glutamate was 1.62 mg/mL, which was 3.6 times higher than that of L-ornithine (*P* < 0.01) and 11 times higher than that of L-aspartic acid (*P* < 0.01). Conversely, the control group exhibited an undetectable low level. These findings confirmed that the three AAs were upregulated metabolites in response to ammonia nitrogen, serving as reservoirs for ammonia nitrogen storage substances in the HJ2 strain. Under ammonia nitrogen treatment, HJ2 effectively converted ammonia nitrogen into L-ornithine, L-glutamate, and L-aspartic acid through their involvement in the arginine pathway. Notably, these three AAs emerged as principal amino acid metabolite products of HJ2 during ammonia nitrogen removal.

**TABLE 1:** Detailed information of amino acids of metabolomic analysis in HJ2 under ammonia nitrogen^*a*^

Name	Formula	KO_ID	Log_2_ FC
L-Glutamate	C_5_H_9_NO_4_	C00025	10.42
L-Valine	C_5_H_11_NO_2_	C00183	4.76
D-Glutamine	C_5_H_10_N_2_O_3_	C00819	4.49
L-Aspartic acid	C_4_H_7_NO_4_	C00049	3.63
L-Histidine	C_6_H_9_N_3_O_2_	C00135	3.58
L-Asparagine	C_4_H_8_N_2_O_3_	C00152	2.66
D-Phenylalanine	C_9_H_11_NO_2_	C02265	2.38
L-Phenylalanine	C_9_H_11_NO_2_	C00079	−0.02
L-Methionine	C_5_H_11_NO_2_S	C00073	1.70
L-Lysine	C_6_H_14_N_2_O_2_	C00047	0.96
L-Tyrosine	C_9_H_11_NO_3_	C00082	0.41
L-Ornithine	C_5_H_12_N_2_O_2_	C00077	1.96
Reduced glutathione	C_10_H_17_N_3_O_6_S	C00051	4.39

^*a*^ KO_ID represented the code number in the KEGG database.

The abundance of various amino acid derivatives also increased, as shown in Table 2. These derivatives were mainly composed of pharmaceutical valuable products and antioxidants. For example, spermidine regulated immunity and protects the heart (46). Hordenine, a dietary component with neuroactivity, had the potential to stimulate hair regeneration (47). L-Dopa served as a precursor for dopamine conversion and was highly effective in the initial stages of Parkinson’s disease (48). Trigonelline had shown resistance to diabetes and provided nerve protection (49). As a coenzyme factor, L-ascorbate and reduced glutathione (GSH) could act as primary antioxidants, maintaining cellular redox stability (50). 6-Hydroxymelatonin contributed to nerve protection and enhances resistance against oxidative stress (51). These findings revealed that toxic ammonia nitrogen tended to synthesize nitrogen-containing metabolites, such as amino acids, amino acid derivatives, and valuable products in the HJ2 strain. HJ2 could promote the transformation of waste nitrogen into nitrogen-containing metabolites and provide beneficial nutrition for aquatic animals.

**TABLE 2:** Detailed information of amino acid derivatives of metabolomic analysis in HJ2 under ammonia nitrogen^*a*^

Name	Formula	KO_ID	Log_2_ FC
Spermidine	C_7_H_19_N_3_	C00315	4.42
Hordenine	C_10_H_15_NO	C06199	1.48
L-Dopa	C_9_H_11_NO_4_	C00355	5.52
Trigonelline	C_7_H_7_NO_2_	C01004	3.49
Ecgonine methyl ester	C_10_H_17_NO_3_	C12448	4.17
Reduced glutathione	C_10_H_17_N_3_O_6_S	C00051	4.39
L-Ascorbate	C_6_H_8_O_6_	C00072	1.43
6-Hydroxymelatonin	C_13_H_16_N_2_O_3_	C05643	3.04
Creatine	C_4_H_9_N_3_O_2_	C00300	1.00
Pipecolic acid	C_6_H_11_NO_2_	C00408	6.10

^*a*^ KO_ID represented the code number in the KEGG database.

### Transformation mechanism of ammonia nitrogen removal of HJ2

**Fig 6:**
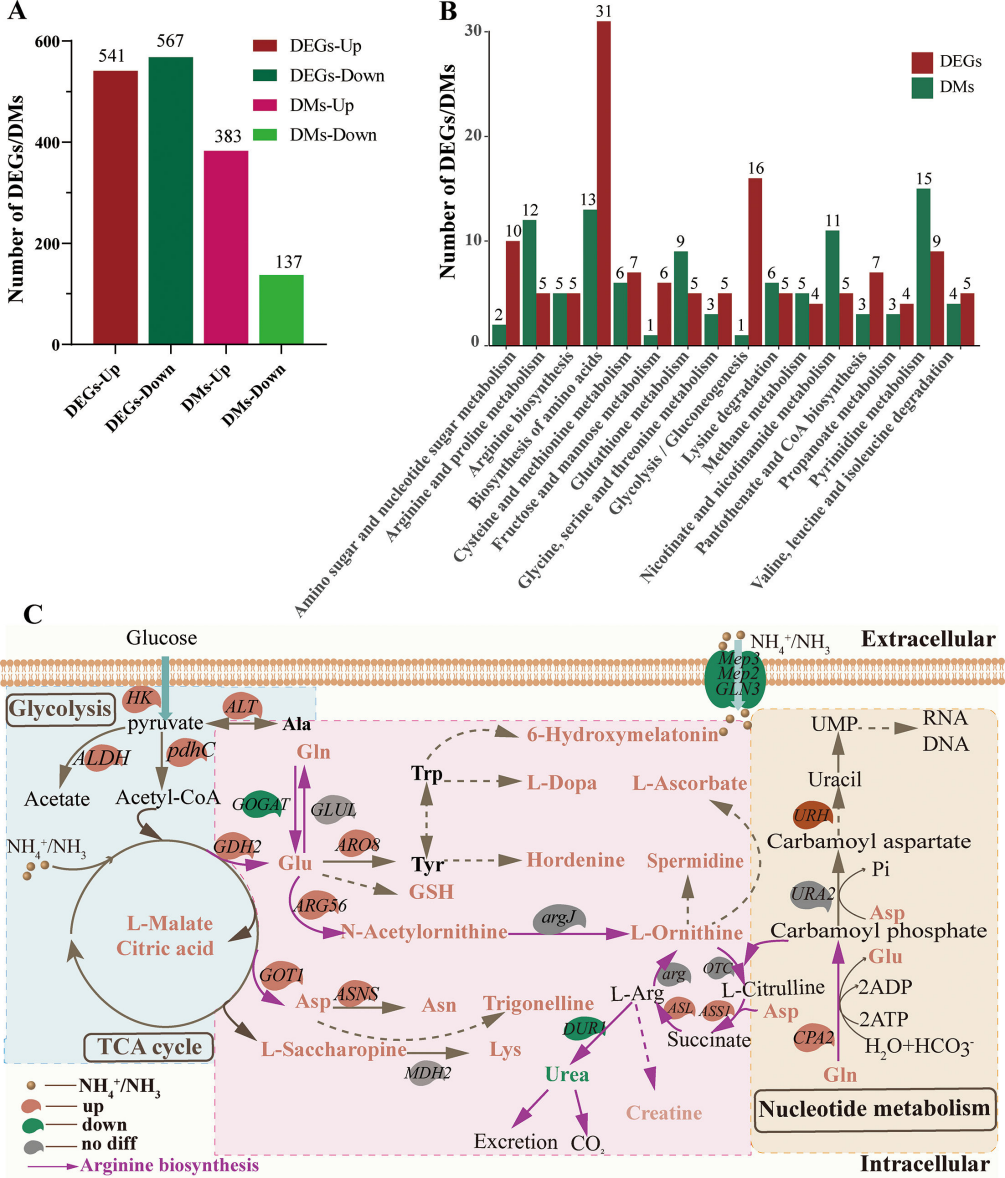
Metabolomics combined with transcriptomics to predict the transformation mechanism of ammonia nitrogen removal in HJ2. (**A**) Statistical diagram of the number distribution of DEGs and DMs. The *y*-axis indicated the number of DEGs/DMs. The *x*-axis indicated the categories of the up-regulated genes, down-regulated genes, up-regulated metabolites, and down-regulated metabolites. (**B**) Statistics of DEGs and DMs enriched in the same KEGG pathway. The ordinate represented the number of DEGs or DMs in a category. The abscissa represented the speciﬁc KEGG pathway. (**C**) Proposed transformation mechanism network of ammonia nitrogen removal in HJ2 by association omics. Genes were indicated in italics. Genes marked in red, green, and gray backgrounds signified up-regulated genes, down-regulated genes, and no significant difference genes, respectively. Red font indicated the up-regulated metabolites, whereas black font denoted the metabolites with no significant difference. For enzyme reactions, the arrows between two metabolites represented the directions of catalytic reactions. Solid arrows represented direct reactions, and dashed arrows indicated indirect reactions. Purple arrows highlighted the arginine synthesis pathways. The blue plate indicated the glycolysis pathway and TCA cycle, the pink plate corresponded to amino acid metabolism, and the orange plate denoted nucleotide metabolism.

As shown in Fig. 6A, under ammonia nitrogen treatment, HJ2 exhibited 541 up-regulated genes and 567 down-regulated genes, accompanied by the modulation of 383 up-regulated metabolites and 137 down-regulated metabolites. Furthermore, Fig. 6B illustrates that the DEGs and associated metabolites were prominently enriched in amino acid metabolism biosynthesis, followed by pyrimidine metabolism and arginine and proline metabolism. These pathways encompassed cellular metabolism involving significant nitrogen compounds, with a particular emphasis on the metabolic processes of synthetic amino acids and pyrimidines.

The transformation mechanism of ammonia nitrogen removal in HJ2 based on transcriptome and metabolome analyses was depicted in Fig. 6C. Under high concentrations of ammonia nitrogen, HJ2 upregulated the ATP synthase gene involved in oxidative phosphorylation metabolism, thereby regulating proton balance and maintaining cellular physiological activities. This process overcame the bioenergy barrier induced by high ammonia conditions, ensuring an energy supply for HJ2’s intracellular metabolism and conferring resistance to ammonia toxicity. Ammonia nitrogen entered the cell through ammonium transporter proteins (Mep2 and Mep3). HJ2 exhibited a notable capacity to produce organic acids, which contributed to the generation of H^+^ required for the conversion of NH_3_ to NH_4_^+^. Through acid production and resistance mechanisms, HJ2 effectively mitigated the detrimental effects caused by ammonia nitrogen alkalization (52). Furthermore, NH_4_^+^ actively participated in cellular metabolism through various ammonia-assimilating enzymes such as GDH, GOT, and GS.

The connection among arginine metabolism, the TCA cycle, and nucleotide metabolism was illustrated in Fig. 6C. The arginine synthesis pathway involved 11 genes, including six up-regulated genes (*GDH2*, *ARG56*, *GOT1*, *CPA2*, *ASL*, and *ASS1*). Glutamate served as the precursor for arginine metabolism by being synthesized from ammonium and α-oxoglutarate by GDH. Subsequently, glutamine, a neutral and non-toxic compound, was synthesized from glutamate. Glutamate and glutamine not only played a crucial role in protein synthesis but also served as primary vehicles for the transportation and storage of ammonia (52, 53). The synthesis of L-ornithine was facilitated by glutamate, which promoted the upregulation of acetyl glutamate kinase (ARG56, K12659, EC: 2.7.2.8). Subsequently, L-ornithine entered the urea cycle pathway. HPLC analysis also revealed the upregulated abundance of L-ornithine, as shown in Fig. 5.

The synthesis of L-ornithine was accomplished through the normal expression of ornithine carbamoyltransferase (OTC, K00611, EC: 2.1.3.3). OTC activity remained unaffected by ammonia toxicity, thereby ensuring the normal operation of the urea cycle. Meanwhile, L-citrulline was synthesized by argininosuccinate synthase (ASS1, K01940, EC: 6.3.4.5). ASS1, as the initial cytoplasmic enzyme involved in the urea cycle, facilitated the condensation of L-citrulline and aspartic acid to produce argininosuccinic acid. The ammonia assimilation pathway catalyzed the synthesis of aspartic acid by GOT. Moreover, within glutamine, aspartic acid served as a precursor for the biosynthesis of carbamoyl phosphate and carbamoyl aspartate through carbamoyl phosphate synthase (CPA2, K01955, EC: 6.3.5.5) and aspartate transcarbamoylase (URA2, K11541, EC: 2.1.3.2). Subsequently, it entered pyrimidine metabolism to facilitate the synthesis of essential genetic material required for cell growth (44).

Under high ammonia nitrogen condition, the up-regulated ASS1 promoted the functioning of the urea cycle. Argininosuccinate was catalyzed by argininosuccinate lyase (ASL, K01755, EC: 4.3.2.1) into L-arginine and fumarate. Fumarate served as an intermediate product of the TCA cycle, leading to the production of L-malate and L-aspartate. Ammonia was further stored as asparagine. Metabolome analysis revealed an upregulated of L-malate (log_2_ FC = 2.71), citric acid (log_2_ FC = 1.87), and L-aspartate (log_2_ FC = 3.63). Conversely, the synthesis of urea by L-arginine exhibited lower toxicity compared with ammonia nitrogen, which was facilitated by the up-regulated of arginase (ARG, K01476, EC: 3.5.3.1) to maintain the normal physiological activities of organisms. The functionality of amino acid metabolism reflected the capability of microorganisms to utilize nitrogen sources for microbial activity and cell synthesis (54). Amino acid metabolism, along with the GDH and GOT ammonia assimilation pathways, played a crucial role in the ammonia nitrogen removal process of the HJ2 strain. Additionally, the urea cycle also contributed an auxiliary transformation function during the ammonia nitrogen removal.

### Ammonia-assimilating enzyme activity tests and the construction of overexpression strains

**Fig 7:**
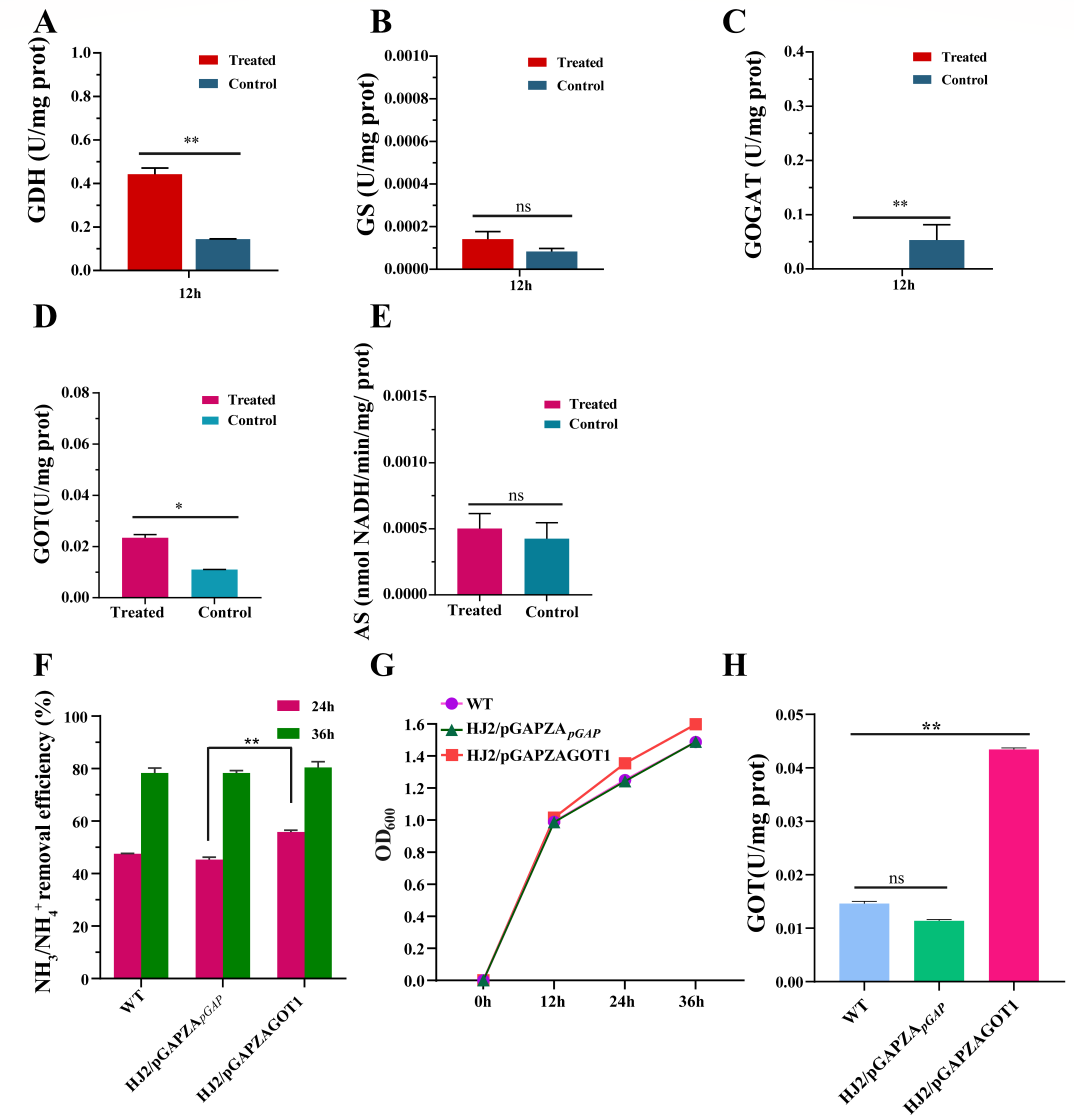
Ammonia-assimilating enzyme activity in HJ2 incubated ammonia nitrogen and ammonia nitrogen removal characteristics of overexpressed strain. Activity of (A) GDH, (B) GS, (C) GOGAT, (D) GOT, and (E) AS of HJ2 incubated in the control and treated groups. The *y*-axis represented the activity of the enzyme. The *x*-axis indicated the time of 12 h of 0 mg/L (control group) and 600 mg/L (treated group) ammonia nitrogen. The data were expressed as mean ± SD. The error bars indicated the standard error of the means for biological triplicates. *P* values were calculated by *t*-test. *P values *<* 0.05, **P values *<* 0.01. (**F**) Ammonia nitrogen removal efficiency of overexpressed strain and other strains. The *y*-axis represented the ammonia nitrogen removal efficiency of the strain. The *x*-axis indicated the incubation time of WT, HJ2/pGAPZA*_pGAP_*, and HJ2/pGAPZAGOT1. The data were expressed as mean ± SD. The error bars indicated the standard error of the means for biological triplicates. *P* values were calculated by *t*-test and one-way ANOVA. *P values *<* 0.05, **P values *<* 0.01. (**G**) Growth of WT, HJ2/pGAPZA*_pGAP_*, and HJ2/pGAPZAGOT1. Purple represented the wild-type strain HJ2, green indicated the strain HJ2/pGAPZA*_pGAP_*, and red denoted the overexpressed strain HJ2/pGAPZAGOT1. The *y*-axis and *x*-axis represented the growth index OD_600_ and different times, respectively. (**H**) Activity of aspartate aminotransferase of WT, HJ2/pGAPZA*_pGAP_*, and HJ2/pGAPZAGOT1. The *y*-axis represented the activity of the GOT enzyme. The *x*-axis indicated the time of 12 h of 600 mg/L ammonia nitrogen. The data were expressed as mean ± SD. The error bars indicated the standard error of the means for biological triplicates. *P* values were calculated by one-way ANOVA. **P values *<* 0.01. Ns represented no significance.

The study investigated variations in enzymatic ammonia assimilation by HJ2 under ammonia nitrogen mediation. The results revealed that the activity concentration of GDH was 0.14 U/mg protein in the control group, whereas it significantly increased to 0.44 U/mg protein in the treated group (*P* < 0.01, Fig. 7A). During ammonia nitrogen removal, the GDH activity of HJ2 was 3.14 times higher than that of the control group. Conversely, there was no significant difference in the GS activity between the two groups (Fig. 7B). Notably, the glutamate synthase (GOGAT) activity in the control group was significantly higher than that in the treated group (Fig. 7C). The activity concentration of GOT in the treated group was 0.023 U/mg protein, which was 2.12 times higher than that in the control group (*P* < 0.01, Fig. 7D). Meanwhile, there was no significant difference in the asparagine synthase (AS) enzyme activity between the two groups (Fig. 7E).

As depicted in Fig. 7F, there was no significant difference in the ammonia nitrogen removal efficiency between the wild-type strain and the control strain HJ2/pGAPZA*_pGAP_* after 24 and 36 h of culture. The result revealed that the empty vector had no significant effect on the ammonia nitrogen removal efficiency of these two strains. However, under the same conditions, the overexpressed strain HJ2/pGAPZAGOT1 showed a significantly enhanced ammonia nitrogen capacity by 8.23% at 24 h (*P* < 0.01) and by 2.1% at 36 h compared with the control strain HJ2/pGAPZA*_pGAP_*. As illustrated in Fig. 7G, under ammonia nitrogen treatment, the overexpressed strain HJ2/pGAPZAGOT1 exhibited superior growth rates compared with the wild-type and control strains. As shown in Fig. 7H, the GOT activities of the wild-type strain HJ2, the control strain HJ2/pGAPZA*_pGAP_*, and the overexpressed strain HJ2/pGAPZAGOT1 were measured after 12 h of ammonia nitrogen treatment. We found no significant difference in the GOT activities between the wild-type strain HJ2 (0.015 U/mg protein) and the control strain HJ2/pGAPZA*_pGAP_* (0.012 U/mg protein). However, the overexpressed strain HJ2/pGAPZAGOT1 exhibited significantly increased activity (0.044 U/mg protein), which was 2.93 (*P* < 0.01) and 3.67 times (*P* < 0.01) higher than that of the wild-type and control strains, respectively.

### Effect of adding exogenous AA on the ammonia nitrogen removal characteristics and growth of HJ2

**Fig 8:**
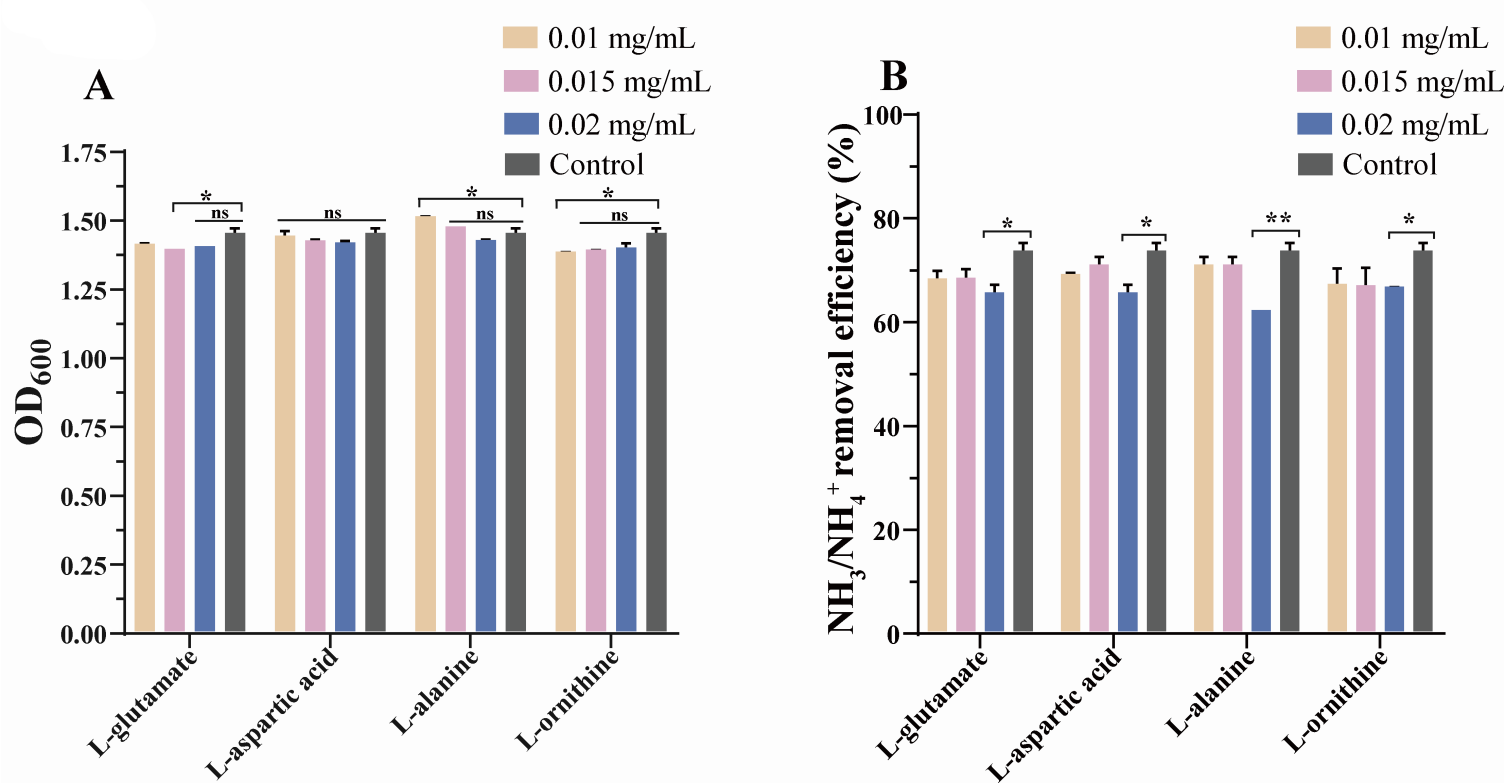
Effect of exogenous amino acids on *P. kudriavzevii* HJ2 incubated for 36 h under 600 mg/L ammonia nitrogen. (**A**) Effect of exogenous amino acids on the growth index of HJ2. The *y*-axis represented the growth index OD_600_. The *x*-axis indicated the categories of exogenous amino acids and the concentration of exogenous amino acids, respectively. (**B**) Effect of exogenous amino acids on the ammonia nitrogen removal efficiency of HJ2. The *y*-axis represented the ammonia nitrogen removal efficiency. The *x*-axis indicated the categories of exogenous amino acids and the concentration of exogenous amino acids, respectively. The data were expressed as mean ± SD. The error bars indicated the standard error of the means for biological triplicates. *P* values were calculated by one-way ANOVA. *P values *<* 0.05, **P values *<* 0.01.

Metabolomic analysis demonstrated that HJ2 could synthesize various amino acids when exposed to high ammonia nitrogen treatment. To investigate the effects of individual amino acids on cell growth and ammonia nitrogen removal efficiency, the study separately added varying concentrations of L-glutamate, L-aspartic acid, L-alanine, and L-ornithine. The results revealed that amino acids could inhibit the growth of the HJ2 strain and reduce its efficiency in removing ammonia nitrogen. As depicted in Fig. 8A, exogenous L-alanine at concentrations of 0.01 mg/mL significantly promoted the growth of HJ2 under ammonia nitrogen treatment, whereas 0.01 mg/mL exogenous L-ornithine and 0.015 mg/mL L-glutamate significantly suppressed HJ2 growth. The ammonia nitrogen removal efficiency, as depicted in Fig. 8B, exhibited the following order from high to low at an exogenous AA concentration of 0.01 mg/mL: L-alanine > L-aspartic acid > L-glutamate > L-ornithine. At 0.015 mg/mL, the sequence was L-alanine = L-aspartic acid > L-glutamate > L-ornithine. When the concentration increased to 0.02 mg/mL, the ammonia nitrogen removal efficiency of HJ2 ranked as L-ornithine > L-glutamate = L-aspartic acid > L-alanine. At 0.02 mg/mL, L-alanine significantly inhibited the ammonia nitrogen removal efficiency of HJ2 (*P* < 0.01). L-glutamate, L-aspartic acid, and L-ornithine also significantly inhibited the ammonia nitrogen removal efficiency (*P* < 0.05) but to a lesser extent compared with L-alanine. These findings indicate that the ammonia nitrogen removal efficiency of HJ2 varied with the concentration of these AAs. The results of the omics analysis and ammonia-assimilating enzyme activity (Fig. 6 and 7) indicated that ammonium was catalyzed by the up-regulated GDH into glutamate. It was reported that glutamate was the main nitrogen donor in cells (26), which was subsequently converted into glutamine by GS in HJ2. Glutamine was then synthesized into carbamoyl phosphate through the up-regulated CPA2. Furthermore, ammonium was converted into aspartic acid by the up-regulated GOT, and aspartic acid could be further converted into asparagine through AS (55). Simultaneously, carbamoyl phosphate and aspartic acid could be catalyzed into N-carbamoyl-L-aspartic acid by URA2, entering the pyrimidine biosynthesis (44). Additionally, alanine could generate glutamate and pyruvate through the up-regulated ALT. In the arginine pathway, glutamate was catalyzed into N-acetylornithine and L-ornithine through the up-regulated ARG56, subsequently entering the urea cycle to produce other nitrogen-containing compounds, such as arginine, spermidine, and L-ascorbate. The results indicated that amino acids played a crucial role in transporting and storing ammonia nitrogen in the HJ2 strain. The addition of exogenous amino acids significantly decreased ammonia nitrogen removal efficiency and inhibited growth to some extent in HJ2. The effect may be attributed to negative feedback inhibition of intracellular ammonia assimilation enzymes induced by the presence of exogenous amino acids (55). Consequently, the ability to synthesize intracellular glutamate, aspartic acid, and other nitrogen-containing compounds (pyrimidine nucleotides) from environmental ammonium by ammonia assimilation-related enzymes was restricted in HJ2, leading to decreased ammonia nitrogen removal efficiency. However, the addition of 0.01 mg/L alanine promoted the growth of HJ2, which would be explored in subsequent experiments.

## DISCUSSION

The presence of ammonia nitrogen in water had emerged as a significant water pollutant, posing a severe threat to the safety of aquatic animals and disrupting the ecological balance of aquatic environments. Consequently, it had been a critical concern in aquaculture (3). Utilization of microorganisms in technology for ammonia nitrogen removal was recognized as the most effective and recommended approach (56). The yeast *P. kudriavzevii* was renowned for its applications in food processing (57). Among its various strains, HJ2 emerged as particularly noteworthy due to its safety profile and promising probiotic attributes, which firmly established it as a highly attractive candidate for the food processing industry (29). Significantly, HJ2 had demonstrated an exceptional survival rate, exceeding 100% in both simulated gastric and intestinal fluids, underscoring its extraordinary resilience in the upper digestive tract. Furthermore, HJ2 exhibited co-aggregation capabilities comparable with those of the probiotic yeast *Saccharomyces boulardii* CNCM I-745, fulfilling the requirements for adherence to intestinal epithelial cells and ensuring robust survival within the upper digestive system. Moreover, HJ2 showed enhanced cholesterol degradation rates and produced higher concentrations of short-chain fatty acids (SCFAs), such as acetic acid and butyric acid (29). HJ2 also exhibited significant antioxidant activity, with an average of 2,2-diphenyl-1-picrylhydrazyl (DPPH) free radical scavenging rate comparable with that of *S. boulardii* CNCM I-745 (58). Importantly, HJ2 outperformed CNCM I-745 in scavenging hydroxyl radicals and consistently demonstrated superior reducing power, both indicative of its stronger antioxidant capacity. To the best of our knowledge, *P. kudriavzevii* had also been shown to possess excellent ammonia nitrogen removal capabilities, further expanding its potential applications. However, the mechanism underlying this process had not yet been fully elucidated (14). Laboratory-based research on microorganisms had provided essential insights into their potential applications for developing specific microbial strain resources (59, 60). However, the reproducibility of optimal culture conditions for particular strains remained challenging in practical industrial applications, owing to the complexity of environmental factors (61). In this study, we investigated the physiological and biochemical parameters, as well as the ammonia nitrogen removal mechanisms of microorganisms, and identified a promising strain as a candidate for treating ammonia nitrogen in aquatic environments. The findings demonstrated that the yeast strain *P. kudriavzevii* HJ2 exhibited remarkable adaptability and efficiency in removing ammonia nitrogen, even under highly acidic, alkaline, or extreme temperature conditions. This result highlightd its potential for commercial application in complex aquatic environments. Moreover, the research contributed to the understanding and development of microbial resources in subtropical marine mangroves. It also served as an important reference for investigating the ammonia nitrogen removal mechanisms of other microorganisms. By exploring the potential of *P. kudriavzevii* in bioaugmentation, the study aimed to enhance the efficiency and adaptability of microbial treatments for nitrogenous wastewater.

The study demonstrated the remarkable efficiency of HJ2 in ammonia nitrogen removal, achieving complete removal (100%) at 300 mg/L ammonia nitrogen and significant removal (73.56%) at 600 mg/L ammonia nitrogen. Compared with *P. kudriavzevii* GW1, HJ2 exhibited a lower capacity for ammonia nitrogen removal (14). However, HJ2 exhibited a higher removal capacity compared with *B. subtilis* Ab03 (30), *B. subtilis* NX-2 (11), and the red yeast *S. pararoseus* Y1 (13). Moreover, HJ2 demonstrated significant ammonia nitrogen removal efficiency across a broad range of pH (2.0–9.0), temperatures (25–45°C), and C/N (5–25). Notably, at lower inoculation concentration levels, HJ2 displayed superior ammonia nitrogen removal efficiency and higher survival rates than strains requiring higher inoculation concentration (27). Furthermore, unlike *B. subtilis* Ab03, *B. subtilis* NX-2, and *S. pararoseus* Y1, which lacked safety reports, the safety and probiotic potential of the yeast strain *P. kudriavzevii* HJ2 had been thoroughly evaluated, making it a promising candidate for ensuring effective water quality management in sustainable farming practices. Regarding carbon sources, HJ2 preferred monosaccharides such as glucose and fructose, which enhanced its ammonia nitrogen removal efficiency, consistent with the findings of Zeng et al. (13). Conversely, HJ2 exhibited the lowest efficiency in removing ammonia nitrogen (9.93%) when using sodium citrate, in contrast to the preference for organic carboxylates by *Stenotrophomonas mailophilia* DQ01 (62). At the same ammonia nitrogen concentration, a significant increase in the cell growth index OD_600_ corresponded to a marked enhancement in the ammonia nitrogen removal efficiency of strain HJ2 within varying culture times (Fig. 1B). Similar to the findings of Xie et al., an increase in the cell growth index was associated with the effective removal of ammonia nitrogen in the strain H1 (34). An unsuitable environment can impair cell growth, ultimately resulting in a reduction of ammonia nitrogen removal efficiency of HJ2. Thus, environmental factors were correlated with both cell growth and ammonia nitrogen removal efficiency.

The gene expression profile and metabolite levels of HJ2 were systematically analyzed using multi-omics techniques (transcriptomics and metabolomics) during ammonia nitrogen treatment. Transcriptome analysis confirmed that the upregulated genes of carbon metabolism, amino acid biosynthesis, and oxidative phosphorylation in the HJ2 strain during ammonia nitrogen treatment supported the synthesis of intracellular macromolecules, facilitating growth and reproduction (38). In arginine metabolism, ammonia nitrogen was converted to glutamate via the up-regulated GDH and then to glutamine via GS. Acetylglutamate kinase catalyzed the synthesis of N-acetylornithine and L-ornithine, initiating the urea cycle. The glutamine synthesis and the urea cycle were effective strategies to deal with ammonia toxicity in cells (3). L-citrulline and L-aspartic acid also entered and promoted the urea cycle. Additionally, ammonium reacted with oxaloacetate to form aspartic acid through the up-regulated aspartate aminotransferase, which was subsequently converted to asparagine. Aspartic acid and glutamine served as nitrogen donors in the pyrimidine metabolic pathway (44). The upregulated gene of pyrimidine metabolism enhanced pyrimidine metabolism, leading to the increased synthesis of upregulated nitrogen-containing metabolites, such as pyrimidine nucleotides, which were essential for RNA and DNA synthesis during the proliferation of HJ2 cells. Additionally, the growth curve data also proved that HJ2 cells exhibited robust growth under high ammonia nitrogen.

Combined with metabolome analysis, the up-regulated of glutamate, aspartic acid, and ornithine simultaneously promoted the production of nitrogen-containing compounds, such as spermidine and trigonelline. The presence of spermidine, a type of polyamine, was found to promote tolerance to high ammonia levels (63). The results of HPLC confirmed the accuracy of the multi-omics findings by demonstrating an increase in amino acid synthesis under high ammonia nitrogen treatment.

In microorganisms, ammonia nitrogen was typically converted into glutamate and aspartic acid by ammonia-assimilating enzymes, subsequently transforming into alanine and ornithine to facilitate the removal of ammonia nitrogen (24, 55). The assessment of enzyme activity enhanced the understanding of the intracellular ammonia assimilation pathway of HJ2, particularly highlighting a significant increase in the activities of GDH and GOT during ammonia nitrogen removal, which improved the efficiency of the process. Experimental results demonstrated that adding exogenous amino acids inhibited the ammonia nitrogen removal capability and growth of HJ2. These findings, along with the results of intracellular total amino acid content, supported the conclusion that amino acids played a critical role in the removal and transformation of ammonia nitrogen in HJ2. HJ2 utilized ammonia nitrogen to synthesize a significant number of amino acids through ammonia assimilation pathways. Over time, amino acids were transformed into other nitrogen-containing metabolites, facilitating protein synthesis and the production of other macromolecules. This process not only removed ammonia nitrogen but also supported the robust growth and proliferation of the HJ2 strain. The arginine pathway was identified as a key metabolic pathway, as it encompasses the ammonia assimilation process.

Previous studies had demonstrated that ammonia-oxidizing bacteria and archaea typically eliminate ammonia nitrogen through aerobic/heterotrophic nitrification and denitrification processes. Ammonia was catalyzed by ammonia monooxygenase (AMO) or methane monooxygenase (MMO) into hydroxylamine, which was subsequently oxidized to nitric oxide (NO) by hydroxylamine oxidoreductase (HAO), and further oxidized to nitrite, ultimately producing nitrogen gas (N₂) (64). In contrast to HJ2, the genome of HJ2 had not been compared with homologous genes for AMO and MMO, possibly because HJ2 was a yeast strain rather than an ammonia-oxidizing microorganism. Additionally, *B. subtilis* NX-2 synthesized the flocculant poly-γ-glutamate from glutamate via γ-PGA synthase to remove ammonia nitrogen (11). The synthesis of poly-γ-glutamate correlated directly with its ability to remove ammonia nitrogen; reduced synthesis results in decreased removal efficiency. The metabolomic profile of HJ2, when treated with ammonia nitrogen, did not indicate the production of poly-γ-glutamate. Instead, HJ2 utilized ammonia nitrogen by synthesizing amino acids and other metabolites through GDH and other ammonia-assimilating enzymes, thereby achieving ammonia nitrogen removal. Furthermore, the ammonia nitrogen removal efficiency of HJ2, following orthogonal optimization, surpassed strain NX-2. Moreover, the strain *Pseudomonas* sp. N31942 had been reported to convert ammonia into nitrate in the absence of hydroxylamine oxidase and nitrite oxidoreductase during the nitrification process (24). In the denitrification process, nitrate or nitrite was reduced to ammonia and subsequently converted into glutamate via the dissimilatory nitrate reduction pathway. Strain N31942 could simultaneously remove ammonia nitrogen through both nitrification and GDH/GS-GOGAT ammonia assimilation pathways. Currently, HJ2 was mainly utilized for ammonia nitrogen removal via ammonia assimilation, while the potential for nitrification would be investigated in subsequent studies. Notably, *P. kudriavzevii* GW1, which exhibited higher ammonia nitrogen removal capability than the HJ2 strain, had yet to reported its ammonia nitrogen removal mechanism for GW1 (14).

The study investigated the remarkable capability of HJ2 to remove ammonia nitrogen through physiological and biochemical experiments. By integrating multi-omics technology and verification experiments, a comprehensive understanding of the relationship between ammonia nitrogen treatment and amino acids metabolism was provided. Transcriptomic and metabolomic analyses identified the genes and metabolites involved in the process. Additionally, this study elucidated the ammonia nitrogen transformation mechanism into non-toxic organic nitrogen in HJ2 through cellular metabolism, thereby promoting its growth and reproduction. These findings offered potential solutions to pollution challenges in aquaculture and helped minimize resource waste. This study also provided theoretical support for the application of microbial agents to treat aquaculture wastewater containing ammonia nitrogen.

## MATERIALS AND METHODS

### Strain, reagents, and media

*P. kudriavzevii* HJ2 was isolated from the subtropical marine mangrove located in the Beibu Gulf of the South China Sea (China General Microbiological Culture Collection Center No. 19897) (28). Glucose, yeast powder, agar powder, peptone, HCl, NaOH, and NaCl—all analytical grade—were purchased from Sigma-Aldrich, Inc. (Darmstadt, Germany). Additionally, K_2_HPO_4_·2H_2_O, MgSO_4_·7H_2_O, MgSO_4_·7H_2_O, EDTA-Na_2_, ZnSO_4_, CaCl_2_, MnCl_2_·4H_2_O, FeSO_4_, H_8_MoN_2_O_4_, CuSO_4_·5H_2_O, CoCl_2_, and Na_3_C_6_H_5_O_7_·2H_2_O were purchased from Novagen (Darmstadt, Germany).

YPD medium (g/L) consisted of glucose (20 g), tryptone (20 g), and yeast extract (10 g). Ammonia nitrogen (AN) medium (g/L) comprised glucose as carbon (C) source (C/N = 25), (NH_4_)_2_SO_4_ (1.43 g), NaCl (1 g), K_2_HPO_4_·2H_2_O (0.5 g), and MgSO_4_·7H_2_O (0.25 g), with 1 mL of trace element solution and an initial pH of 7.0. Trace element solution was composed of the following (g/L): EDTA-Na_2_ (10 g), ZnSO_4_ (1.2 g), CaCl_2_ (1.5 g), MnCl_2_·4H_2_O (1 g), FeSO_4_ (2 g), H_8_MoN_2_O_4_ (1 g), CuSO_4_·5H_2_O (1 g), and CoCl_2_ (1 g) (65). Each treatment was conducted in triplicate.

### Physicochemical characteristics and ammonia nitrogen removal of HJ2

The initial culture of *P. kudriavzevii* HJ2 was activated through incubation in YPD agar plates (28). Subsequently, the seed liquid was subjected to cultivation for 24 h on AN medium under varying conditions, including ammonia nitrogen concentration (100–600 mg/L), temperature (25°C, 30°C, 35°C, 37°C, 40°C, and 45°C), inoculation concentrations (0.5%, 1%, 1.5%, 2%, 4%, 6%, and 8%), initial solution pH (2.0, 3.0, 5.0, 7.0, and 9.0, adjusted with 1 M HCl), C/N (5, 10, 15, 20, and 25), and different carbon sources (glucose, fructose, sucrose, maltose, starch, and sodium citrate). Additionally, HJ2 was cultured on AN medium for different time points (12, 24, 36, 48, and 72 h), and the ammonia nitrogen concentration of AN medium of temperature, inoculation concentrations, initial solution pH, C/N, and carbon sources was 300 mg/L. The cell growth index OD_600_ of HJ2 was measured by spectrometer (BioTek Instruments, Inc., Winooski, VT, USA). Through single-factor experiments, an orthogonal design containing three factors and three levels was employed to optimize the NH_3_/NH_4_^+^ removal efficiency conditions under 600 mg/L ammonia nitrogen. The specified inoculation concentrations were established at 0.5%, 1.0%, and 1.5%. The cultivation times were 24, 36, and 48 h, and the temperatures were 30°C, 35°C, and 40°C. The design scheme of the orthogonal design was shown in Table S5.

The growth curve of HJ2 under 0 (control group) and 600 mg/L (treated group) ammonia nitrogen concentration was evaluated by measuring the OD_600_ value. The strain was harvested through centrifugation at 8,000 × *g* for 10 min and was examined by scanning electron microscopy (Thermo Electron Instruments Co., Ltd., Czech Republic).

### Intracellular total AA content of *P. kudriavzevii* HJ2 under ammonia nitrogen treatment

HJ2 was incubated for 12 h under different conditions: 0 (control group) and 600 mg/L (treated group) ammonia nitrogen concentration, inoculated with 0.5% (vol/vol) seed solution, and cultured at 35℃ with 200 rpm. Yeast solution was collected in 50 mL for 12, 24, 36, and 48 h, followed by centrifugation at 4℃ and 6,000 × *g* for 15 min to collect yeast precipitate. Total AAs were determined using an AA Detection Kit (Solarbio Technology Co., Ltd., Beijing, China) (66).

### Analytical methods

The fermentation broth from HJ2 was centrifuged at 12,000 × *g* for 2 min to collect the supernatant. The ammonia nitrogen concentration was measured by using the nitrogen ammonia salicylate TNT kit (HACH Co., Ltd., USA) and DR900 multi-parameter portable colorimeter (HACH Co., Ltd., USA) (13). A separate AN medium was inoculated with sterile double-distilled water as the negative control. pH was measured using a pH meter (PHS-3 C, Sanxin, China). The OD_600_ values were determined by turbidimetric detection.

### Transcriptome analysis of HJ2

HJ2 was incubated for 12 h under ammonia nitrogen concentrations of 0 and 600 mg/L at 35°C with 200 rpm. Total RNA was extracted within three biological replicates in each group. RNA integrity was assessed using the RNA Nano 6000 Assay Kit on the Bioanalyzer 2100 system (Agilent Technologies, Santa Clara, CA, USA). Library construction and quality inspection followed the methods provided by Novogene (Beijing, China). Libraries were pooled based on the effective concentration and the target offline data volume targets for Illumina Sequencing-By-Synthesis by Novogene (Beijing, China). The original data were filtered to obtain clean data to ensure the quality and reliability of data analysis. Trinity software was used for transcript assembly, and splicing was assessed using Corset hierarchical clustering. The quality of slices was evaluated using BUSCO software on Trinity.fasta, unigene.fa, and cluster.fasta. The gene function was annotated based on the following databases: Nr (NCBI non-redundant protein sequences), Nt (NCBI non-redundant nucleotide sequences), Pfam (Protein family), KOG/COG (Clusters of Orthologous Groups of proteins), Swiss-Prot (a manually annotated and reviewed protein sequence database), KO (KEGG Ortholog database), and GO (Gene Ontology). Differential expression analysis between the two groups was conducted using the DESeq2 R package (1.20.0). *P* values were adjusted using the Benjamini–Hochberg approach to control the false discovery rate. The gene or transcript with *P*_adj_ < 0.05 and |log_2_ fold change| ≥1 was considered as DEGs/transcripts. DEG enrichment of GO and KEGG pathways was performed using the clusterProfiler R package.

### Metabolome analysis of HJ2

The culturable supernatant of HJ2 treated with ammonia for 0 and 600 mg/L for 24 h of fermentation was utilized to assess the metabolic activity. Each group consisted of six biological replicates for metabolite determination. The samples (100 µL) were transferred to Ep tubes and were mixed with pre-chilled 80% methanol by thorough vortexing. The supernatant was subjected to analysis using the LC–MS/MS system (67). UHPLC–MS/MS analyses were performed using a Vanquish UHPLC system (Thermo Fisher, Dreieich, Germany) coupled with an Orbitrap Q Exactive HF mass spectrometer (Thermo Fisher) at Novogene (Beijing, China). The raw data files were processed using Compound Discoverer 3.1 (CD3.1, Thermo Fisher) to perform peak alignment, peak picking, and quantitation analyses for each metabolite. The normalized data were used to predict the molecular formula based on the additive ions, molecular ion peaks, and fragment ions. The peaks were matched with the mzCloud (https://www.mzcloud.org/), mzVault, and MassList databases to obtain accurate qualitative and quantitative results. Statistical analyses were performed using the R software (R version R-3.4.3), Python (Python 2.7.6 version), and CentOS (CentOS release 6.6). When data were not normally distributed, normal transformations were attempted using the area normalization method. Metabolites were annotated using the KEGG (https://www.genome.jp/kegg/pathway.html), HMDB (https://hmdb.ca/metabolites), and LIPIDMaps databases (http://www.lipidmaps.org/). PCA was performed using metaX (68). Univariate analysis (*t*-test) assessed the statistical significance (*P* value). Metabolites with a VIP > 1, *P* value < 0.05, and |log_2_ fold change| ≥1 were considered DMs. The functions of these metabolites and metabolic pathways were analyzed using the KEGG database. Metabolic pathway enrichment of DMs was determined based on ratios x/n > y/N, indicating pathway enrichment. Additionally, metabolic pathways with a *P* value < 0.05 were considered statistically significant in terms of enrichment.

### Co-expression network analysis of transcriptome and metabolome

Correlation analysis was performed using Pearson’s correlation coefficient in the R language mixOmics package to calculate the correlation coefficient R^2^ and the *P* value of DEGs and DMs. All identified genes and metabolites from the control and treated groups were mapped to the KEGG database to obtain their common pathway information and conduct statistical analyses.

### RT-qPCR and HPLC validation for DEGs and DMs

On the basis of the results from the combined transcriptome and metabolome analysis, seven DEGs and three DMs were selected for RT-qPCR analysis and HPLC verification. The size of primers and products are shown in Table S2. Total RNA from HJ2 was extracted using the universal total RNA rapid extraction kit (YFX Biotechnology Co., Ltd., Nanjing, China). First-strand cDNA was synthesized using the YfxScript 1st strand cDNA Synthesis Kit. RT-qPCR was carried out at the final concentrations of 20–25 ng/µL template DNA, 250 ng/µL forward primer, and 250 ng/µL reverse primer. We added appropriate 2× SYBR Green Fast qPCR Master Mixture/−LR/HR and adjusted with RNase-free H_2_O. The reaction conditions included initial heating at 95°C for 2 min, followed by 40 cycles of denaturation at 95°C for 15 s, annealing/extension at 60°C for 30 s, and a melting curve from 63°C to 95°C with an increase of 0.3°C/10 s. Real-time fluorescence quantitative PCR was conducted on a LightCycler480 (Roche Co., Ltd., Basel, Switzerland) using Software Release 1.5.1.62 SP3. Relative quantitation of the selected genes was calculated using the 2*^-ΔΔCt^* method.

Additionally, the concentrations of AA (L-glutamate, L-ornithine, and L-aspartate) in the supernatants from 0 (control group) to 600 mg/L (treated group) of NH_3_/NH_4_^+^ were assessed by HPLC pre-column derivatization (69). The AAs were measured using an Alliance liquid chromatograph (Waters E2695, USA), with a Poroshell120 SB-Aq 4.6 × 250 mm, 4 µm column (Agilent Technologies Inc., USA), and a column temperature of 45℃. The flow rate was set at 1 mL/min with an injection volume of 5 µL. Detection was performed at a wavelength of 254 nm.

### Ammonia-assimilating enzyme activity tests

In the study, 0.5% seed solution was inoculated into the control group of 0 mg/L and the treated group of 600 mg/L ammonia nitrogen medium. The cultures were incubated in a shaker incubator at a constant temperature of 35°C and 200 rpm for 12 h. Thereafter, cells were centrifuged at 6,000 × *g* for 10 min to collect the cell precipitate. The precipitate was washed three times with 10 mL of 50 mM PBS, and 0.2 g of the precipitate was transferred into a new centrifuge tube as a sample. The yeast total protein of HJ2 strain under different ammonia nitrogen condition was extracted and the concentration was determined by the yeast total protein extraction kit (Solarbio Technology Co., Ltd., Beijing, China) and BCA protein content determination kit (Solarbio Technology Co., Ltd., Beijing, China) (70). The activities of ammonia-assimilating enzymes were detected by using a GDH activity assay kit (Solarbio Technology Co., Ltd., Beijing, China), a GS activity assay kit (Solarbio Technology Co., Ltd., Beijing, China), a GOGAT activity assay kit (Solarbio Technology Co., Ltd., Beijing, China), a GOT activity assay kit (Solarbio Technology Co., Ltd., Beijing, China), and an AS activity detection kit (Jining Industrial Co., Ltd., Shanghai, China).

### Construction of overexpression strain HJ2/pGAPZAGOT1

For overexpressing *GOT1* in *P. kudriavzevii* HJ2, the commercial plasmid pGAPZA (Invitrogen, Waltham, MA, USA) was used to construct the integration expression vector pGAPZAGOT1. The primers used for plasmid construction were listed in Table S6. The bleomycin gene was used as a positive selection marker against the antibiotic zeocin. The GAPDH promoter and gene *GOT1* were synthesized based on the genome of HJ2, with the GAPDH promoter influencing the expression of *GOT1*. The plasmid pGAPZA*_pGAP_* was linearized via reverse amplification PCR using primers 1F/1R. The gene *GOT1* was synthesized via PCR using primers 2F/2R. The fragment products were directionally and simultaneously ligated to obtain pGAPZAGOT1 using ClonExpress MultiS One Step Cloning Kit (Vazyme Biotech, Nanjing, China). The construct was then introduced into *P. kudriavzevii* HJ2 by electroporation using Gene-Pulser II (Bio-Rad, Hercules, CA, USA) at 2.5 kV, 25 µF, and 200 Ω. After electroporation, the cells were incubated in 1 mL of YPD for 3 h and were plated on solid YPD medium containing 1800 μg/mL zeocin. The transformants were selected by PCR using primers 3F/3R to obtain the overexpression strain HJ2/pGAPZAGOT1. An empty vector pGAPZA*_pGAP_* with the GAPDH promoter but without the gene *GOT1* insert was constructed and transformed into *P. kudriavzevii* HJ2. The transformants were selected on selective solid YPD medium containing 1,800 μg/mL zeocin and selected by PCR using primers 4F/4R to obtain control strains HJ2/pGAPZA*_pGAP_*. The overexpression plasmid map and electrophoresis gel maps of its construction process were listed in Fig. S5.

### Effect of adding exogenous amino acids on the ammonia nitrogen removal characteristics and growth of *P. kudriavzevii* HJ2

A stock solution (2.5 mg/mL) of L-glutamate, L-aspartic acid, L-alanine, and L-ornithine was prepared. In ammonia nitrogen medium (NH_3_/NH_4_^+^ = 600 mg/L) inoculated with 0.5% (vol/vol) HJ2, the final concentrations of AAs (0.01, 0.015, and 0.02 mg/mL) were achieved by adding AA stock solution. The control group consisted of an ammonia nitrogen medium without any added AAs. The culture medium was incubated at 35°C for 36 h at 200 rpm. The growth index OD_600_ of HJ2 was measured. Yeast suspension was collected by centrifugation at 12,000 × *g* for 2 min. The ammonia nitrogen concentration in the supernatant was measured using the nitrogen ammonia salicylate TNT kit (HACH Co., Ltd., USA) and DR900 multi-parameter portable colorimeter (HACH Co., Ltd., USA).

### Statistical analysis

All data values were presented as mean ± standard deviation (SD). Data were processed with GraphPad Prism 8.0.2. Statistical analysis and graphical representation were conducted using *t*-test and one-way analysis of variance (ANOVA) to evaluate significant differences. Significance was indicated by lowercase letters (a, b, c, etc.) or asterisks (*). *P* values of *<*0.05 (*) and *<*0.01 (**) were considered to indicate significant differences and highly significant differences, respectively. All figures were made with GraphPad Prism 8.0.2, Adobe Photoshop (CC 2017), and Adobe Illustrator (2021).

## Data Availability

The authors confirm that the data supporting the findings of this study are available within the article and its supplemental material. The transcriptomic data of *P. kudriavzevii* HJ2 under ammonia nitrogen treatment have been deposited in the NCBI Sequence Read Archive (SRA) (accession no. PRJNA1242297).

## SUPPLEMENTAL MATERIAL

Supplemental material aem.02211-24-s0001.xls Tables S1 to S6; Fig. S1 to S5.

ASM does not own the copyrights to Supplemental Material that may be linked to, or accessed through, an article. The authors have granted ASM a non-exclusive, world-wide license to publish the Supplemental Material files. Please contact the corresponding author directly for reuse.
